# Mesenchymal Transition and Dissemination of Cancer Cells Is Driven by Myeloid-Derived Suppressor Cells Infiltrating the Primary Tumor

**DOI:** 10.1371/journal.pbio.1001162

**Published:** 2011-09-27

**Authors:** Benjamin Toh, Xiaojie Wang, Jo Keeble, Wen Jing Sim, Karen Khoo, Wing-Cheong Wong, Masashi Kato, Armelle Prevost-Blondel, Jean-Paul Thiery, Jean-Pierre Abastado

**Affiliations:** 1 Singapore Immunology Network, BMSI, A-STAR, Singapore; 2 Institute for Molecular and Cellular Biology, BMSI, A-STAR, Singapore; 3 Bioinformatics Institute, BMSI, A-STAR, Singapore; 4 College of Life and Health Sciences, Chubu University, Aichi, Japan; 5 Institut Cochin, Université Paris Descartes, CNRS UMR 8104, Paris, France; 6 Cancer Science Institute, National University of Singapore, Singapore; London Research Institute, United Kingdom

## Abstract

In order to metastasize, cancer cells need to acquire a motile phenotype. Previously, development of this phenotype was thought to rely on the acquisition of selected, random mutations and thus would occur late in cancer progression. However, recent studies show that cancer cells disseminate early, implying the existence of a different, faster route to the metastatic motile phenotype. Using a spontaneous murine model of melanoma, we show that a subset of bone marrow-derived immune cells (myeloid-derived suppressor cells or MDSC) preferentially infiltrates the primary tumor and actively promotes cancer cell dissemination by inducing epithelial-mesenchymal transition (EMT). CXCL5 is the main chemokine attracting MDSC to the primary tumor. In vitro assay using purified MDSC showed that TGF-β, EGF, and HGF signaling pathways are all used by MDSC to induce EMT in cancer cells. These findings explain how cancer cells acquire a motile phenotype so early and provide a mechanistic explanation for the long recognized link between inflammation and cancer progression.

## Author Summary

Cancer progression has been depicted as a linear process, during which the incipient cancer cell sequentially accumulates mutations that confer the ability to metastasize. However, recent studies show that cancer cells disseminate early, before such mutations can accumulate, implying the existence of a different, faster route to the metastatic phenotype. Using a mouse model of melanoma, we show that the primary tumor attracts a subset of immune cells that actively promote cancer cell motility, dissemination, and metastasis. These tumor-infiltrating immune cells do so by reactivating a cellular program (mesenchymal transition) used by melanocytes during their development to colonize the skin, and also believed to be an essential step in cancer cell dissemination and metastasis. Once the melanoma cells migrate out of the primary tumor, they can lapse back to their original phenotype and lose their migratory potential. This transient phenotypic switch may accelerate carcinogenesis and participate in the plasticity of cancer. It explains how cancer cells might spread to other organs even before the original tumor is detected. In addition to the evidence gleaned from our mouse melanoma model, we show that these immune cells induce typical features of epithelial-mesechymal transition in both melanoma and bladder human cell lines when examined in culture dishes. These findings provide an underlying mechanism for the long-recognized link between inflammation and cancer progression.

## Introduction

Tumor metastasis is the primary cause of death by cancer [1]. Metastasis is a multistep process in which cancer cells derived from the primary tumor migrate to regional or distant sites where they re-initiate tumor development [2]. Until recently, the first step of metastasis, (i.e. tumor cell dissemination) was thought to be a late event in cancer progression [3]. This time-lag was presumably needed to allow selected cancer cells to accumulate the additional mutations required for motility. However, recent work, including that of our laboratory, has challenged this paradigm. In fact, cancer cells disseminate even before diagnosis of the primary tumor [4]–[6], and so a different, faster mechanism must be driving the development of the motile phenotype.

Tumors do not consist of a homogeneous population of cells; rather they are a composite of cancer cells, mesenchymal and endothelial cells, and immune cell populations. Among these immune cells are the myeloid cells, which are generating increasing interest as having a dynamic influence on tumor growth. The link between cancer progression and infiltration with myeloid cells was recognized by R. Virchow in the late 19^th^ century [7]–[11]. Infiltration with myeloid cells is usually associated with less favorable clinical outcomes. During the past decade several distinct subsets of tumor-infiltrating myeloid cells have been described [12], among which CD11b^+^Gr1^+^ MDSC have drawn attention for having a role in cancer progression [13]–[16]. MDSC, which can be further divided into monocytic (Mo-) MDSC and granulocytic (PMN-) MDSC, accumulate in most malignant murine and human tumors [17]–[19]. These cells have been shown to favor cancer progression by dampening anti-tumor immune responses, promoting angiogenesis, and creating a pre-metastatic environment [20]–[22].

RETAAD mice are transgenic for the activated *RET* oncogene, which is specifically expressed in skin and eye melanocytes. RETAAD mice spontaneously develop uveal melanomas that are clinically detectable (exophthalmos) by 4 to 8 wk of age. In fact, microscopic eye tumors can be detected as early as 10 d after birth and cancer cells disseminate from the primary eye tumor throughout the body by 3 wk of age [5],[23]. Disseminated cancer cells can be monitored in the eye draining lymph node and in the lung by measuring ectopic expression of *Dct* (a specific marker of melanocytes) or *RET*. The disseminated cancer cells remain dormant for months before developing into cutaneous and then visceral metastases. Previous work showed that most tumors developing in a given mouse share a common clonal origin [5]. The stepwise evolution of melanoma in RETAAD mice mimics the natural history of disease progression in cancer patients [24], underlining the suitability of this model for dissecting the role of immune cells in the process of metastasis.

Although melanocytes are not of epithelial origin, metastatic melanoma cells undergo morphological changes resulting in decreased intercellular adhesion and increased cell motility, a process that morphologically and mechanistically resembles epithelial-mesenchymal transition (EMT) [25]. EMT is a trans-differentiation process occurring during embryonic development and in carcinoma progression [26]. In the present study we used RETAAD mice to examine the presence and role of MDSC in melanoma metastasis. Interestingly, we found that PMN-MDSC preferentially accumulate in the primary tumor where they induce cancer cell EMT. PMN-MDSC favor the rapid acquisition of a motile phenotype by cancer cells resulting in multinodular growth of the primary tumor, dissemination throughout the body and distant metastasis.

## Results

### Gradual Accumulation of PMN-MDSC in Tumor-Bearing Mice

RETAAD mice develop uveal melanoma that metastasizes to the skin and visceral organs. Tumor development at distant sites usually takes more than 6 mo. As previously described in other tumor models [18],[19], we observed an increase in CD11b^+^Gr1^+^ cells in both the spleen and blood during tumor progression (Figure S1A and B, respectively). This increase begins as the primary tumor starts to cause the eye to bulge (exophthalmos) and peaks once distant metastases become palpable. As shown in Figure S1C, purified CD11b^+^Gr1^+^ cells strongly inhibited antigen-specific T cell proliferation in vitro and therefore represent bona fide MDSC.

### PMN-MDSC Preferentially Accumulate in Primary Tumors

**Figure 1:**
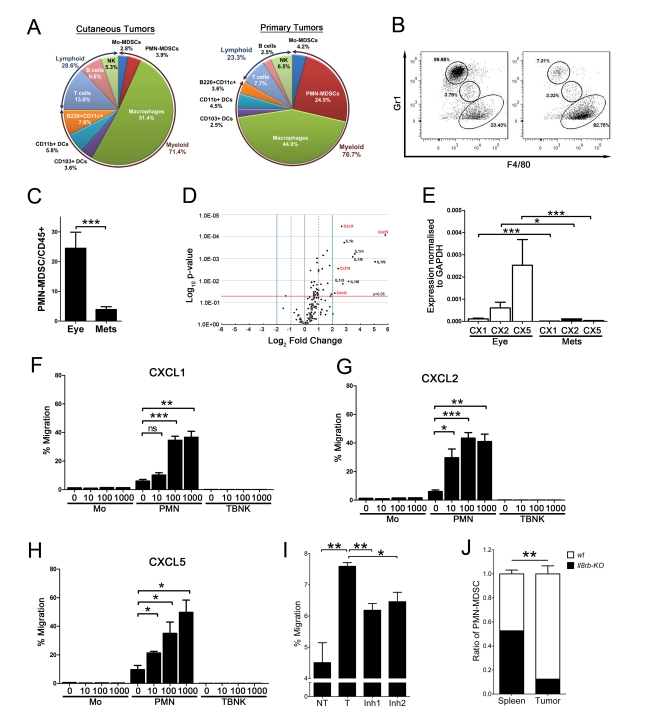
CXCR2 ligands attract PMN-MDSC to the primary tumor. (A) Quantification of the subsets of immune cells infiltrating primary tumors and cutaneous metastases. Data were obtained by flow cytometry from 20 cutaneous and 13 eye tumors. Numbers show the frequencies of each subset among CD45^+^ cells. Gating strategy for immune cell subsets is illustrated in Figure S1D. (B) Preferential accumulation of PMN-MDSC (Gr1^hi^ F4/80^−^) in primary tumors. Composition of the primary tumor and a cutaneous cheek tumor from the same mouse. Cells are gated on CD45^+^CD11c^−^CD11b^+^DAPI^−^ tumor cells. (C) Frequency of PMN-MDSC in primary (*n* = 13) and cutaneous (*n* = 20) tumors. Bars represent mean ± SEM. ***p value <0.0001, two-tailed unpaired Student’s *t* test. (D) Repertoire of chemokines and cytokines differentially expressed in primary tumors and metastases. Fold changes represent the log_2_ ratio of gene expression measured by qRT-PCR in primary tumors (*n* = 11) and in the metastatic tumors (*n* = 11). *p* values were calculated by two-tailed paired Student’s *t* test. (E) Relative expression of CXCL1 (CX1), CXCL2 (CX2), and CXCL5 (CX5) in 11 primary tumors and 11 metastases measured by qRT-PCR. *p<0.05, ***p<0.001. (F–H) CXCL1 (F), CXCL2 (G), and CXCL5 (H) induce PMN-MDSC chemotaxis in vitro, but have no effect on monocytes or T, B, and NK lymphocytes. *x*-axis represents the amount of chemokine added in ng/ml. Percentage migration was calculated as (number of migrated cells) ×100/(total cells added per well). Data are from three independent experiments carried out in duplicates. Bars represent mean ± SEM *p<0.05, **p<0.01, ***p<0.001, two-tailed *t* test. (I) Inhibition of CXCR2 reduces PMN-MDSC migration in vitro. PMN-MDSC were treated 1 h before and during the migration assay with CXCR2 inhibitors—Inh1 (SB265610) and Inh2 (SB225002). Percentage migration was calculated as (number of migrated cells) ×100/(total cells added per well). Data are from two independent experiments carried out in duplicates. Bars represent mean ± SEM *p<0.05, **p<0.01, two-tailed *t* test. (J) CXCR2 is required for PMN-MDSC accumulation into the primary tumor. Equal numbers of bone marrow cells from Rosa mT/mG reporter mice expressing tdTomato (used as fluorescently tagged wild-type [*wt*] cells) and *Il8rb-KO* mice expressing GFP were adoptively transferred into tumor-bearing RETAAD mice. The graph shows the contribution of each genotype to donor-derived PMN-MDSC present in the primary tumor and spleen 18 h after transfer. Data are from four mice. Bars represent mean ± SEM *p<0.05, **p<0.01, two-tailed *t* test.

CD11b^+^Gr1^+^ MDSC are a heterogeneous population of myeloid cells. To further characterize MDSC in the RETAAD model, immune infiltrates of primary tumors and cutaneous metastases were analyzed. CD45^+^ immune cells represented on average 2.7% (95 CI = 1.9%–3.5%) of the total cells within a tumor, in good agreement with published data on spontaneous melanoma models [27] and uveal melanoma patients [28]. Among immune cells infiltrating cutaneous tumors (Figure 1A), lymphoid cells represented 28.6% (95 CI = 20.1%–37.1%). The tumor-infiltrating myeloid cell population comprised CD103^+^CD11c^hi^ dendritic cells (3.6%, 95 CI = 2.5%–4.7%), CD11b^+^CD11c^hi^ dendritic cells (5.8%, 95 CI = 4.2%–7.4%), B220^+^CD11c^+^ plasmacytoid dendritic cells (7.6%, 95 CI = 3.3%–11.0%), CD11b^+^Gr1^−^F4/80^+^ macrophages (51.4%, 95 CI = 42%–61%) and CD11b^+^Gr1^+^ MDSC (6.7%, 95 CI = 3.6%–9.8%). MDSC could be further sub-divided into CD11b^+^Gr1^hi^ F4/80^−^ PMN-MDSC and CD11b^+^Gr1^int^F4/80^lo^ Mo-MDSC (Figure 1 and Figure S1D). Interestingly, while most subsets of immune cells were equally represented in primary tumors and cutaneous metastases (Figure 1A), PMN-MDSC were 5 times more abundant in primary tumors (Figure 1B and 1C, 24.5%±1.5% of immune cells versus 3.9%±0.2%; two-tailed *t* test *p*< 0.0001). In order to understand the significance of this finding, we decided to examine the factors controlling the preferential accumulation of PMN-MDSC in the primary tumor and to investigate the role of these cells.

### Primary Tumors Express PMN-MDSC Chemoattractants

To identify potential mediators of the preferential accumulation of PMN-MDSC in primary tumors, the expression of 148 inflammatory genes including chemokines, inflammatory cytokines, and their corresponding receptors was analyzed using low density qRT-PCR arrays. When primary tumors and cutaneous metastases from the same mice were compared, 21 genes were identified as differentially expressed (fold change >2; *p*<0.05), of which 20 were more highly expressed in the primary tumor than in the corresponding skin tumor (Figure 1D and Table S1). The most differentially expressed genes (fold change >4 and *p*<0.05) were members of the IL-1 families and, interestingly, four chemokines: CCL19, CXCL1, CXCL2, and CXCL5. For example, the average expression of CXCL5 was 56-fold higher in primary tumors than in cutaneous metastases (*p* = 8.38×10^−5^). Transcriptome analysis of purified tumor-infiltrating immune cells indicated that only PMN-MDSC express *Il8rb* (also known as *CXCR2*), the receptor for CXCL1, CXCL2, and CXCL5 (Figure S2). Furthermore, PMN-MDSC express CXCL1 and CXCL2 while tumors express CXCL5. We therefore tested the capacity of these three chemokines to attract immune cells from tumor-bearing RETAAD mice in vitro. As shown in Figure 1F–H, CXCL1, CXCL2, and CXCL5 are indeed potent attractants for PMN-MDSC, but not monocytes or lymphocytes. Moreover, RETAAD tumor cells attracted PMN-MDSC in vitro and this attraction was significantly reduced when PMN-MDSC were treated with either SB265610 or SB225002, two specific inhibitors of CXCR2 (Figure 1I). To establish the importance of CXCR2 ligands in the recruitment of PMN-MDSC to the primary tumors, bone marrow cells from Rosa mT/mG reporter mice expressing tdTomato (used as fluorescently tagged wild-type [*wt*] cells) and CXCR2 knockout mice expressing GFP (*IL8rb-KO* crossed with mice expressing EGFP under the lysozyme M promoter [*Il8rb-KO*]) were mixed at a 1∶1 ratio and adoptively transferred into tumor-bearing RETAAD mice. After 18 h, primary tumors were analyzed by flow cytometry for Gr1^hi^tdTomato^+^ and Gr1^hi^GFP^+^ tumor-infiltrating PMN-MDSC. *Wt* PMN-MDSC were 7-fold more abundant than PMN-MDSC from *Il8rb-KO* mice (Figure 1J). This preferential migration was specific for the primary tumor as equal proportions of *wt* and *Il8rb-KO* PMN-MDSC migrated to the spleen. This shows that expression of CXCR2 is necessary for PMN-MDSC migration into the primary tumor in vivo. Of the three CXCR2 ligands, CXCL5 is the most highly expressed in primary tumors (Figure 1E) and is therefore likely to be the major mediator of the preferential recruitment of PMN-MDSC to the primary tumor. In summary, primary tumors express unique chemokines that specifically attract PMN-MDSC.

### PMN-MDSC Favor the Growth of the Primary Tumor

**Figure 2:**
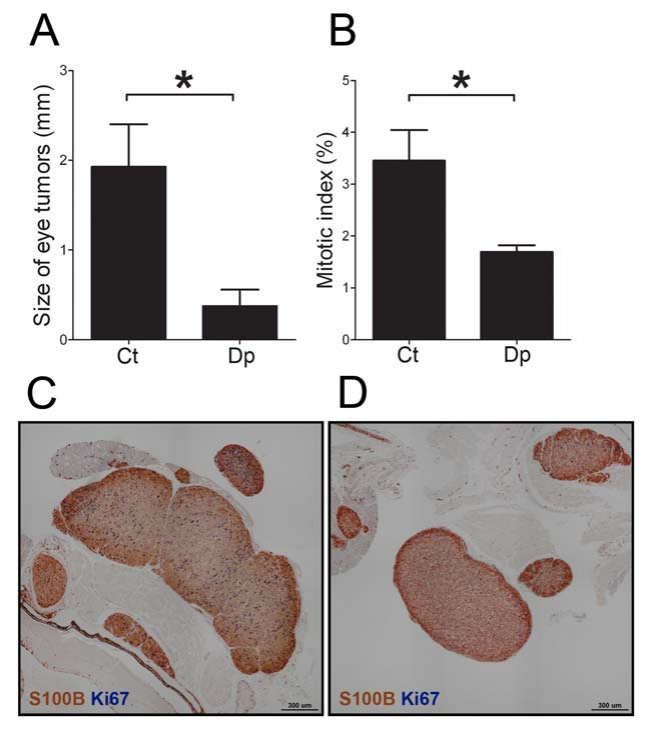
PMN-MDSC favor tumor cell proliferation in the primary tumor. (A) Five-week-old mice were depleted of PMN-MDSC by bi-weekly injection of anti-Ly6G antibody (NIMP-R14) (depletion scheme B). At 20 wk of age, mice were euthanized and the size of the primary tumors was measured. Results are from a total of 22 tumors derived from four depleted mice (Dp) and seven littermates injected with a control antibody (Ct). (B–D) One-week-old mice were depleted of PMN-MDSC by bi-weekly injection of anti-Ly6G antibody (depletion scheme A). At 7 wk of age, mice were culled and eye tumors were analyzed by two-color immunohistochemistry using S100B- (brown) and Ki67- (blue) specific antibodies. (B) Comparison of the mitotic indices measured in 172 nodules taken from eight depleted mice (Dp) and 130 nodules from seven control littermates (Ct). Panels C and D show representative examples of tumors from control and depleted mice. Bars in panels A and B represent mean ± SEM. *p value <0.05, two-tailed unpaired Student’s *t* test.

To gain insight into the role of PMN-MDSC in primary tumors, these cells were depleted using anti-Ly6G antibody NIMP-R14 [29]. The first injection of antibodies was given as soon as the primary tumor became visible (5 wk of age), then repeated twice a week, until the age of 20 wk when tumors were analyzed by flow cytometry and immunohistochemistry (Figure S3A, scheme B). Antibody treatment ablated PMN-MDSC (CD11b^+^Gr1^hi^) in the primary tumor and spleen (determined at the time of sacrifice) and in the blood for the duration of the experiment (Figure S3B). Importantly, the monocytic subset of MDSC (Mo-MDSC, CD11b^+^Gr1^lo^F4/80^lo^) was not affected by the depletion, whereas there was a slight increase in macrophage number (CD11b^+^Gr1^−^F4/80^+^) in the primary tumors (Figure S3B). Interestingly, depletion of PMN-MDSC resulted in reduced growth of the primary tumors (Figure 2A) but not cutaneous tumors (Figure S4). At 20 wk of age, the mean diameter of primary tumors was 4.7 times smaller in depleted mice compared to mice treated with an isotype control antibody (mean diameter 1.9±0.5 mm versus 0.4±0.2 mm; two-tailed *t* test *p* = 0.027). Enhanced angiogenesis was unlikely to explain the effect of PMN-MDSC on tumor growth since no significant difference in the density of blood vessels or CD45^−^CD31^+^ endothelial cells existed between PMN-MDSC-depleted and control tumors (Figure S5A and B). In fact, there was a trend towards an increased density of CD45^−^CD31^+^ cells in the PMN-MDSC depleted mice. Taken together this shows that PMN-MDSC favor the growth of the primary tumor.

### PMN-MDSC Promote Cancer Cell Proliferation in the Primary Tumor

To elucidate the mechanism by which PMN-MDSC favor growth of the primary tumor, we focused on the early steps of tumorigenesis. The depletion experiment was repeated starting at 1 wk of age, before any eye abnormality was detectable, and the primary tumors were analyzed at 7 wk of age (Figure S3A, scheme A). At this age macroscopic primary tumors are rarely detectable, and when microscopic tumors were analyzed by immunohistochemistry, no difference was observed in the size of the lesion developing in treated and control mice (Figure S6). However immunohistochemistry revealed that depletion of PMN-MDSC results in a reduced frequency of proliferating cancer cells, identified by double labeling with S100B- and Ki67-specific antibodies (Figure 2C and D). As shown in Figure 2B, at 7 wk of age, primary tumors exhibited a mitotic index of 3.5%±0.6% that was reduced to 1.7%±0.1% (*p* = 0.01) in PMN-MDSC depleted mice. We calculated that such a 2-fold difference in the mitotic indices would translate into a reduction in tumor diameter of just under 2-fold at 7 wk and 4-fold at 20 wk, showing good agreement with the observed 4.7-fold (Figure 2A and Text S1). Taken together, MDSC depletion experiments showed that these cells favor cancer cell proliferation (detected at 7 wk) leading to the development of bigger primary tumors (at 20 wk).

### PMN-MDSC Secrete Soluble Factors That Promote Cancer Cell Proliferation In Vitro

**Figure 3:**
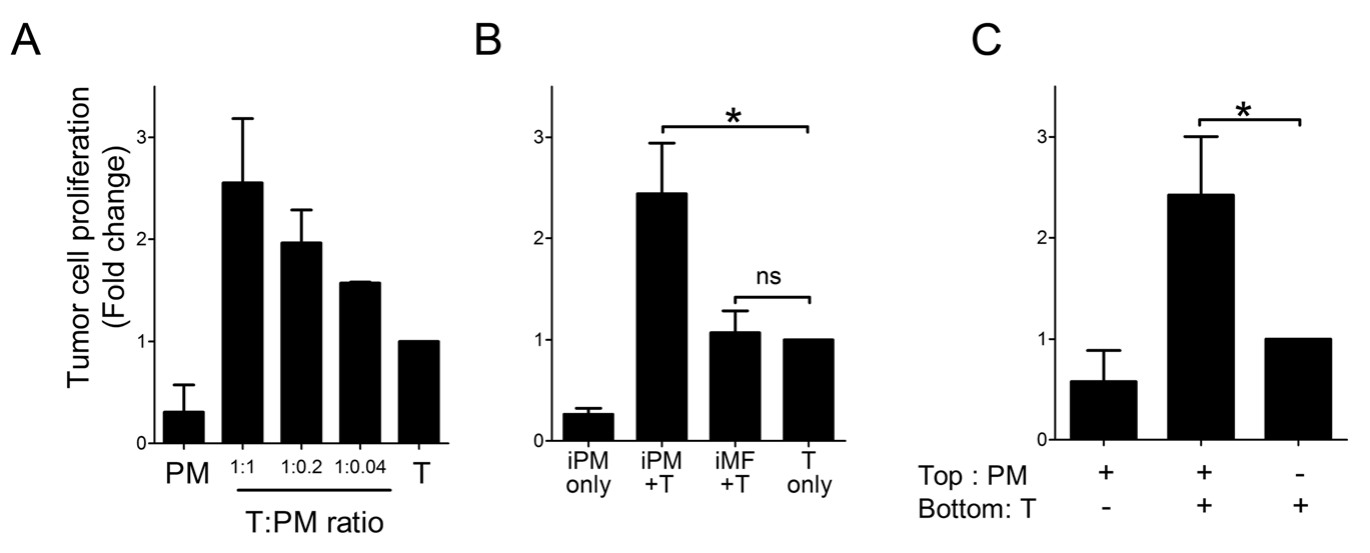
PMN-MDSC favor tumor cell proliferation in vitro. (A) Melan-ret cells (5×10^3^ cells per well) were cultured with increasing concentrations of PMN-MDSC purified from tumor-bearing RETAAD mice (12-wk-old) for 48 h. Proliferation of tumor cells was assessed by [^3^H]-thymidine incorporation. PM, PMN-MDSC only; T:PM, ratio of tumor cells to PMN-MDSC; T, tumor cells only. (B) Irradiated PMN-MDSC but not macrophages induce Melan-ret tumor cell proliferation. Macrophages and PMN-MDSC were irradiated at 2,000 rads before co-culture with tumor cells for 48 h. iPM, Irradiated PMN-MDSC; iMF, Irradiated macrophages; T, tumor cells. (C) PMN-MDSC-induced proliferation does not require direct contact. PMN-MDSC and tumor cells were plated in the top and bottom wells of transwell inserts (pore size=0.4 µm; Millipore), respectively. PMN-MDSC cultured in the upper chamber were able to induce proliferation of Melan-ret tumors cells in the bottom chamber after 48 h. Data are from four independent experiments carried out in duplicates. Bars represent the average fold change relative to tumor cell only ± SEM. *p value <0.05, two-tailed paired Student’s *t* test.

To determine whether the effect of PMN-MDSC on cancer cell proliferation was direct or required additional stromal components, in vitro co-culture experiments were performed. Melan-ret cells, a cell line derived from a RET tumor, were co-cultured for 48 h with PMN-MDSC purified from tumor-bearing RETAAD mice. Cancer cell proliferation was measured in vitro by [^3^H]-thymidine incorporation. Addition of PMN-MDSC resulted in increased proliferation of the cancer cells in a dose-dependent manner (Figure 3A). This effect was not due to proliferation of PMN-MDSC since even irradiated PMN-MDSC induced cancer cell proliferation (Figure 3B, *p* = 0.035). Importantly this was specific to PMN-MDSC, as co-culture with F4/80^+^ cells purified from tumor-bearing RETAAD mice did not enhance cancer cell proliferation (Figure 3B). Furthermore, it did not require direct contact between the PMN-MDSC and the cancer cells as increased proliferation was also observed when PMN-MDSC were separated from the cancer cells by a porous membrane (Figure 3C, *p* = 0.025). Taken together, these results show that PMN-MDSC secrete soluble mediators that directly promote cancer cell proliferation.

### PMN-MDSC Favor Multinodular Development of Primary Tumors

**Figure 4:**
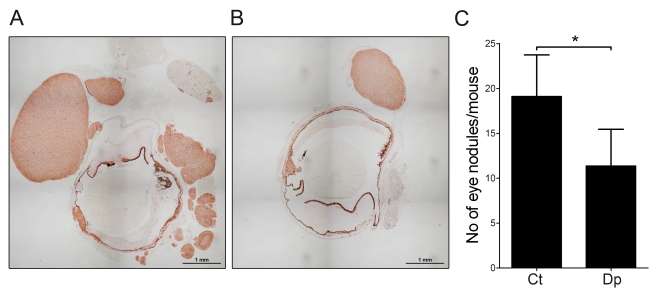
PMN-MDSC favor multinodular growth of the primary tumor. (A and B) Multinodular eye tumors from control (A) and PMN-MDSC depleted (B) mice stained with anti-S100B antibody (depletion scheme A). (C) Number of nodules per mouse. Results are from 11 depleted (depletion scheme A) mice (Dp) and 10 littermates injected with control antibody (Ct). *p value <0.05, two-tailed paired *t* test.

In the RETAAD model, primary tumors of the eye display a multinodular structure. Given that in this model, tumors from the same mouse share a common clonal origin, this multinodular structure was suggestive of early acquisition of a motile phenotype [5],[30]–[32]. We wondered whether PMN-MDSC played any role in the migration of cancer cells to the tumor periphery. Primary tumor morphology was analyzed by histology in 7-wk-old mice that had been depleted of PMN-MDSC from 1 wk of age onward. As shown in Figure 4, primary tumors from depleted mice (*n* = 11) had smaller numbers of nodules compared to control mice (*n* = 11). The average number of ocular nodules was 11±4 in the depleted mice and 19±5 in the controls (two-tailed Wilcoxon *p* = 0.04). This result shows that PMN-MDSC play a role in development of the multinodular structure of primary eye tumors by favoring the migration of melanoma cells to the tumor periphery.

### PMN-MDSC Induce Cancer Cell Dissemination to Regional and Distant Sites

**Figure 5:**
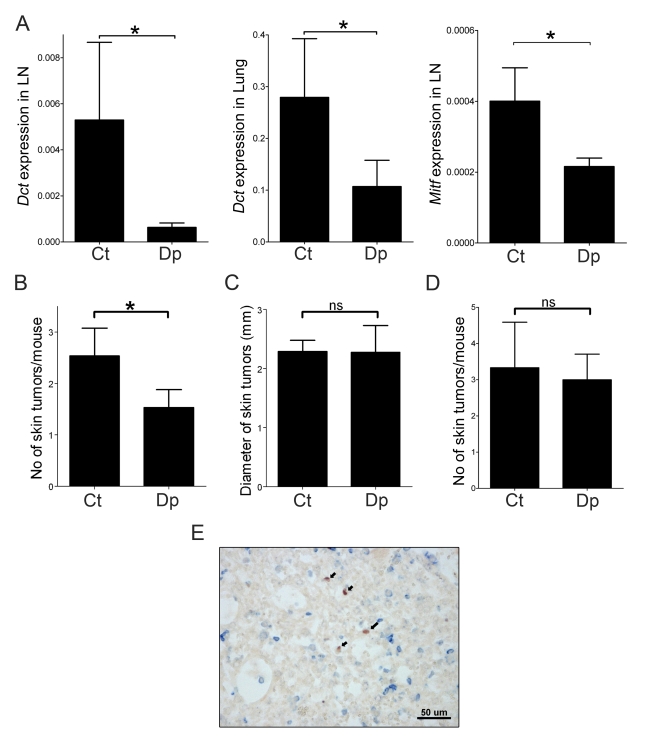
PMN-MDSC stimulate tumor cell dissemination and metastatic outgrowth. (A) Dissemination of tumor cells to the draining mandibular lymph nodes (from 12 control and 20 depleted mice) and lungs (from 11 control and 11 depleted mice) was assessed by measuring ectopic expression of *Dct and Mitf* by qRT-PCR. The graph shows the relative gene expression normalized to *GAPDH* in mice injected with the control (Ct) or the anti-Ly6G antibody (Dp) (depletion scheme A). Bars represent mean ± SEM. *p<0.05, two-tailed *t* test. (B) Decreased number of cutaneous tumors in mice depleted from PMN-MDSC. Data are from 11 control and 11 PMN-MDSC-depleted mice (depletion scheme A). Bars represent mean ± SEM. *p<0.05, two-tailed Wilcoxon matched-pairs test. (C) Depletion of PMN-MDSC does not affect the size of cutaneous tumors. The depletion scheme A was used, but similar results were obtained with depletion scheme B (Figure S4). Bars represent mean ± SEM. *ns*, non-significant. (D) Delayed depletion of PMN-MDSC has no effect on the incidence of cutaneous metastases. Depletion scheme B was used. Data are from four control (Ct) and six PMN-MDSC-depleted (Dp) mice. Bars represent mean ± SEM. *ns*, non-significant. (E) Disseminated melanoma cells found in the lungs of 7-wk-old mice are mostly individual cells. Red, S100B; blue, CD45.

Given that PMN-MDSC favor the migration of melanoma cells to the tumor periphery, we wondered whether they also promoted cancer cell dissemination to more distant sites, such as the eye-draining mandibular lymph nodes or the lungs. Daupachrome tautomerase (*Dct*) is specifically expressed by melanocytic cells and ectopic expression of *Dct* represents a sensitive surrogate marker of cancer cell dissemination [5]. We therefore compared *Dct* expression in the eye-draining mandibular lymph nodes and lungs of 7-wk-old, PMN-MDSC-depleted and control mice. As shown in Figure 5A, there was an 8.4-fold decrease (*p* = 0.03) in *Dct* expression in the mandibular lymph node draining the primary tumor (left panel) and a 2.6-fold decrease (*p* = 0.02) in *Dct* expression in the lungs (central panel) of depleted mice compared to the isotype control treated animals. A similar difference (1.8-fold; *p* = 0.01) was also observed in *Mitf* (a melanocyte-specific transcription factor) expression in the mandibular lymph node (Figure 5A, right panel). In addition, immunohistochemical analysis of lung sections showed that, in 7-wk-old mice, tumor cells present in lungs were mostly individual cells, implying that the increased *Dct* expression was not due to larger micro-metastases (Figure 5E). This finding rather shows that, in addition to promoting peri-tumoral dissemination, PMN-MDSC favor metastasis to regional and distant sites.

### PMN-MDSC Favor Metastatic Outgrowth

Cancer cell dissemination to the skin of RETAAD mice cannot be easily measured because skin contains melanocytes that express *Dct* and *Mitf* under physiological conditions. However, we were able to quantify the number of macroscopic cutaneous tumors in 7-wk-old mice depleted of PMN-MDSC from 1 wk of age and control mice. As shown in Figure 5B, there was a 40% reduction (*p* = 0.01, Wilcoxon matched-pairs test; two-tailed) in the number of cutaneous tumors in mice depleted of PMN-MDSC between 1 and 7 wk of age. The distribution of tumor sizes was similar between treated and control animals (Figure 5C), consistent with the low percentage of PMN-MDSC in cutaneous tumors. These data show that PMN-MDSC infiltrating the primary tumor promote cancer cell dissemination to cutaneous sites. This increased influx of disseminated cancer cells results in an increased number of metastases without affecting the average size of tumor.

By comparing the pattern of mutations found in primary tumors and metastases, we previously showed that metastases derive from cancer cells that disseminate early [5]. We therefore predicted that if PMN-MDSC play a critical role in cancer cell dissemination, depleting these cells after cancer cells have already colonized the skin should have little effect on the onset of cutaneous metastases. Indeed, delaying the onset of PMN-MDSC depletion until 5 wk of age (Figure S3A, Scheme B) restored the incidence of cutaneous metastases to that of control mice (Figure 5D). These data indicate that most cutaneous tumors derive from cancer cells that disseminate before 5 wk of age and that PMN-MDSC act at an early stage of melanoma development.

### PMN-MDSC Induce Cancer Cell EMT In Vitro

**Figure 6:**
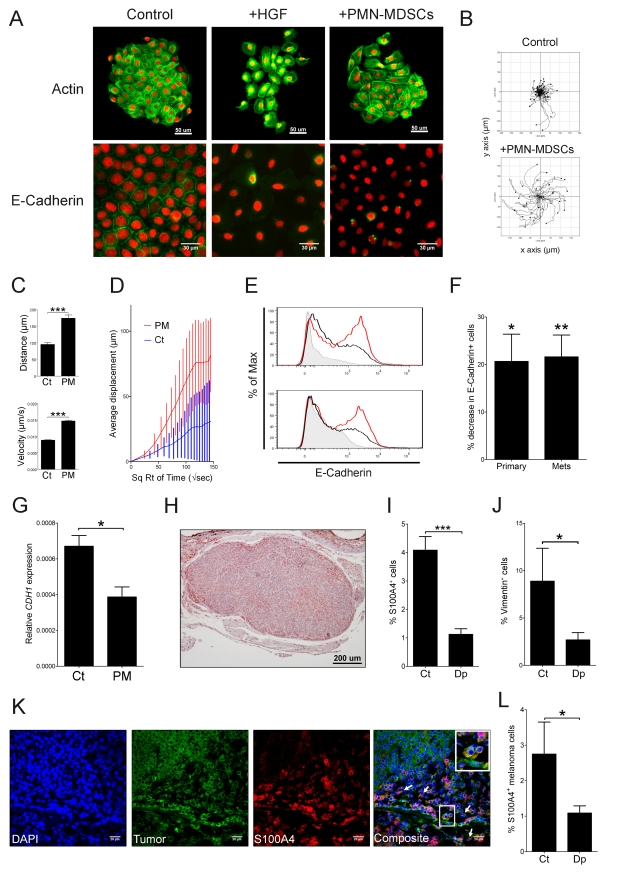
PMN-MDSC induce EMT in vitro and in vivo. (A) PMN-MDSC (1×10^4^ cells per well) were purified from 12-wk-old RETAAD mice and co-cultured with NBT-II cells for 24 h. HGF (5 ng/ml) was added as a positive control for induction of EMT. Green, Actin (upper panel) or E-Cadherin (lower panel); red, H2b (nuclear stain). Images are representative of three independent experiments. (B–D) Individual colonies of NBT-II cells were tracked for 6 h (during hours 12–18) out of the 22 h captured. Tracks of 59 cells from the control and 32 cells co-cultured with PMN-MDSC (1×10^4^ cells per well) were analyzed. Panel B illustrates the individual tracks of all the cells in the colony. Panels C and D show an increase in distance traveled, speed, and displacement of NBT-II cells when co-cultured with PMN-MDSC. PM, PMN-MDSC; Ct, Control. Bars represent mean ± SEM. ***p<0.0001, two-tailed *t* test. (E) Primary RETAAD tumors (top panel) or cutaneous metastases (bottom panel) were dissociated and co-cultured for 24 h with (black line) or without (red line) PMN-MDSC purified from the same mouse. Data are gated on CD45^−^ live cells. Filled histograms: isotype control. (F) Co-culture for 24 h with PMN-MDSC (as described in Figure 6E) induced a decrease in the number of tumor cells expressing E-Cadherin. Data are from five primary tumors and five cutaneous metastases in three independent experiments. Bars represent the mean decrease in the number of live CD45^−^ E-Cadherin^+^ cells ± SEM *p<0.05, **p<0.01, two-tailed *t* test. (G) PMN-MDSC decrease *CDH1* gene expression in vitro. Human melanoma cell line, 888mel, was co-cultured with PMN-MDSC purified from a tumor-bearing RETAAD mouse. Cells were washed and harvested after 24 h. Data are from three independent experiments. Bars represent mean ± SEM *p<0.05. (H–I) Staining for S100A4 in primary eye tumor nodules. Panel H is a representative staining of primary tumors for S100A4. Red, S100A4; blue, Heamatoxylin (Nuclear stain). Panel I shows a decrease in the percentage of S100A4^+^ cells in the tumors depleted from PMN-MDSC (depletion scheme A). Dp, PMN-MDSC depleted mice; Ct, Control mice. Bars represent mean ± SEM of 176 control and 87 PMN-MDSC depleted tumor nodules. ***p<0.0001, two-tailed *t* test. (J) Decreased percentage of vimentin^+^ cells in tumors depleted from PMN-MDSC (depletion scheme A). Dp, PMN-MDSC depleted mice; Ct, Control mice. Bars represent mean ± SEM of 14 pairs of primary tumors. *p<0.05, two-tailed paired *t* test. (K) The majority of S100A4^+^ cells express melanoma specific antigens. Eye tumors were co-stained for melanoma antigens (HMB45 and MelanA/MART-1) and S100A4. White arrows indicate S100A4^+^ melanoma cells exhibiting a fusiform morphology. Note the different morphologies of the double-stained mesenchymal-like cells and single-stained ones. Blue, DAPI (nucleus); green, HMB45/DT101/BC199 (melanoma); red, S100A4. (L) Decreased percentage of S100A4+ melanoma cells in tumors depleted from PMN-MDSC (depletion scheme A). Double positive cells stained for HMB45/MART-1 and S100A4 were quantified from three depleted (Dp) and three control (Ct) mice.

To further investigate the mechanisms by which PMN-MDSC induce cancer cell dissemination and metastasis, we analyzed the morphology and motility of NBT-II bladder carcinoma cells co-cultured with PMN-MDSC purified from tumor-bearing RETAAD mice. NBT-II cells were chosen as reporter cells because they are known to undergo EMT in response to several different stimuli [33]–[35]. Following co-culture with PMN-MDSC for 24 h, NBT-II cells acquired a mesenchymal morphology (Figure 6A). Actin microfilaments redistributed from the membrane cortex to the basal surface, indicating loss of cell-cell contact. Moreover, NBT-II cells exhibited numerous thin filopodia and cell surface expression of E-Cadherin, a classical marker of epithelial cells, was down-regulated (Figure 6A). Similar changes were observed when NBT-II cells were treated with Hepatocyte Growth Factor (HGF), a known inducer of EMT [36]. To assess motility, NBT-II cells were tracked for 6 h using time-lapse video microscopy (Videos S1–S3). As seen in Figure 6B–D, NBT-II cells co-cultured with PMN-MDSC travelled significantly further (174±9 µm versus 95±5 µm, *p* = 6.7×10^−12^), with an increased average velocity (53.0±0.9 µm/h versus 32.1±0.4 µm/h, *p* = 2.8×10^−106^) resulting in a larger total displacement (80.0 ± 5.5 µm versus 29.3 ±3.9 µm, *p* = 3.1×10^−11^). To assess the generality of this observation, PMN-MDSC were co-cultured with dissociated tumor cells from RETAAD mice. After 24 h, down-regulation of cell surface E-Cadherin was observed in tumor cells derived from both primary tumors (21% reduction; *p* = 0.023) and cutaneous metastases (22% reduction; *p* = 0.005; Figure 6E–F). Down-modulation of E-Cadherin gene expression was also observed in the human melanoma cell line 888mel after co-culture with PMN-MDSC purified from RETAAD mice (Figure 6G; 42% reduction: *p* = 0.025). Taken together, these data demonstrate that upon co-culture with PMN-MDSC, cancer cells undergo morphological, behavioral, and phenotypic changes typical of EMT.

### PMN-MDSC Induce Cancer Cell EMT In Vivo

These in vitro findings led us to predict that in vivo depletion of PMN-MDSC would affect the frequency of cancer cells undergoing EMT in the primary tumor. S100A4 (also known as Fsp1) is a small Ca^2+^ binding protein involved in EMT and cell motility [37]–[39]. We measured the number of S100A4^+^ melanoma cells in primary tumors. Interestingly, S100A4^+^ cells were found specifically in the periphery of tumor nodules (Figure 6H). Importantly primary tumors from mice depleted of PMN-MDSC between 1 and 7 wk of age possessed a 3.6-fold lower density of S100A4^+^ cells (Figure 6I; *p* = 3.42×10^−8^ in two-tailed *t* test). Similarly, a 3.3-fold decrease was seen in vimentin expression (Figure 6J; *p* = 0.035 in paired two-tailed Wilcoxon-test). Closer characterization of S100A4^+^ cells in primary tumors showed that there were indeed delaminating melanoma cells expressing HMB45 and MART-1 antigens and exhibiting a fusiform morphology (white arrows; Figure 6K). The number of S100A4^+^ cells expressing melanoma differentiation antigens was reduced in tumors depleted from PMN-MDSC (Figure 6L). This observation indicates that PMN-MDSC play a crucial role in EMT induction in melanoma cells in vivo.

### PMN-MDSC Induce EMT through Multiple Pathways

**Figure 7:**
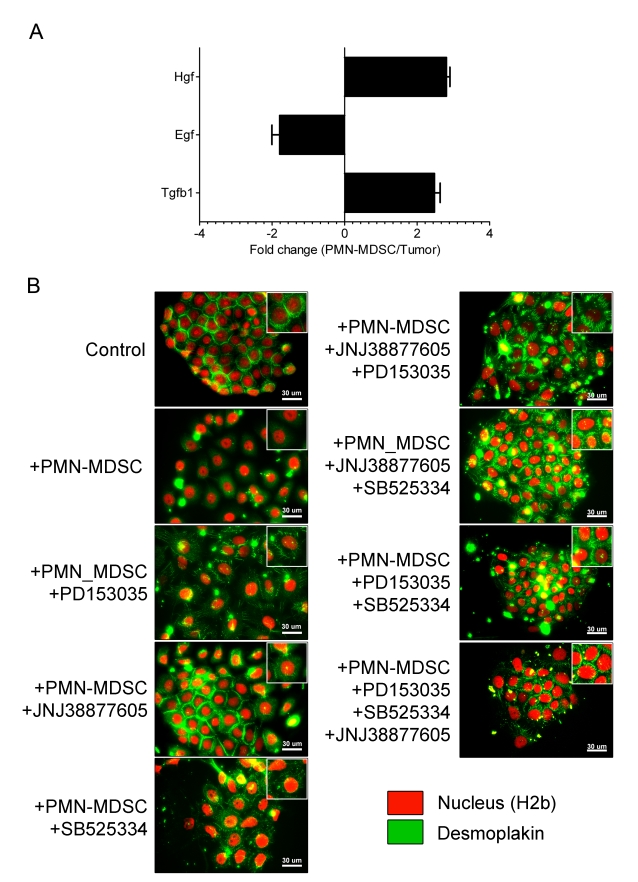
Inducers of EMT. (A) Expression of Hgf, Egf, and Tgfb1 genes in RETAAD tumors analyzed by qRT-PCR. Hgf and Tgfb1 are preferentially expressed in PMN-MDSC, while Egf is preferentially expressed in tumor cells. Data are from four individual experiments using sorted fractions of PMN-MDSC and melanoma cells. Bars represent mean ± SD. (B) NBT-II cells (100 cells per well) were plated for 4 d to allow for colony growth and were pre-treated for 24 h with inhibitors before the addition of PMN-MDSC. After co-culture with PMN-MDSCs in the presence of inhibitors, cells were fixed and stained with anti-rat desmoplakin. Green, Desmoplakin; red, H2b (nuclear stain). PD153035 – EGFR inhibitor, JNJ38877605 – c-met (HGFR) inhibitor, and SB525334 – TGF-βR1 inhibitor. Images are representative of three independent experiments.

To identify the molecular pathways involved in PMN-MDSC-induced EMT, the various cell subsets present in RETAAD tumors were sorted by flow cytometry before performing transcriptome analysis and searching for known inducers of EMT. As shown in Figure 7A, PMN-MDSC were found to express Hepatocyte Growth Factor (HGF) and Transforming Growth Factor-β1 (TGF-β1), while cancer cells were the main source of Epidermal Growth Factor (EGF). To determine whether these factors played any role in PMN-MDSC-induced EMT, NBT-II cells were pre-treated with various inhibitors before co-culture with PMN-MDSC. After 24 h, NBT-II cells were stained with desmoplakin to visualize the desmosomes that are typically found at the junctions between epithelial cells (Figure 7B). Addition of PMN-MDSC to untreated NBT-II cells resulted in the complete dissociation of the desmosomal complexes at the cell surface and their translocation into the cytoplasm. Cell scattering was strongly reduced by pre-treatment of NBT-II cells with SB525334, a specific inhibitor of the receptor for TGF receptor 1 (TGF-βR1). Nevertheless, most desmoplakin junctions were lost. In contrast, most cells treated with JNJ38877605, a specific inhibitor of the receptor for HGF (HGFR), retain desmoplakin at cell-cell junctions. Further inhibition of EMT was observed when NBT-II cells were pretreated with SB525334 together with JNJ38877605 and/or PD153035, a specific inhibitor of the receptor for EGF (EGFR). Pre-treatment of NBT-II cells with all three inhibitors completely reversed the effect of PMN-MDSC. Similar results were obtained when a different set of inhibitors was used (Figure S7). Taken together, these results demonstrate that TGF-β1, EGF, and HGF play a crucial role in EMT induction by PMN-MDSC.

### Discussion

Recent data suggest that primary tumor cells acquire a metastatic phenotype during the early stages of cancer progression [5],[40]. This hypothesis led to a conundrum, as the previously accepted theory of random accumulation of mutations leading to the metastatic phenotype was unable to explain such rapid cancer cell dissemination. Here we show that a particular subset of immune cells preferentially infiltrates primary tumors, induces EMT in primary tumor cells, and promotes cancer cell dissemination. Therefore, in our mouse model of melanoma, acquisition of a motile phenotype by cancer cells is instructed by tumor-infiltrating MDSC and occurs early in the development of the primary tumor.

MDSC accumulate in the tumors, spleen, and blood of tumor-bearing mice [19],[41]. In the RETAAD model, we uncovered a preferential recruitment of PMN-MDSC to primary tumors compared to cutaneous metastases, which correlated with a more inflammatory microenvironment. Importantly the chemokines CCL19, CXCL1, CXCL2, and CXCL5 were significantly more highly expressed in primary tumors. CXCL1, CXCL2, and CXCL5 are known chemoattractants for neutrophils [42],[43] and, as shown here for the first time to our knowledge, potent attractants of PMN-MDSC as well. CXCR2, the receptor for CXCL2, CXCL5, and CXCL1, is specifically expressed on PMN-MDSC purified from RETAAD tumors (Figure S2). Pharmaceutical inhibition of CXCR2 reduced PMN-MDSC attraction by melanoma cells in vitro and genetic deletion of CXCR2 impaired their recruitment to the primary tumor in vivo. Since CXCL5 is the most highly expressed of the three CXCR2 ligands, it is the prime candidate for mediating the preferential attraction of PMN-MDSC to the tumor. In addition, since CXCL1 and CXCL2 are expressed by PMN-MDSC purified from RETAAD tumors, we propose that these two chemokines provide a positive feedback loop that further amplifies the accumulation of PMN-MDSC in primary tumors. Of note, CXCL1 and IL-8, the human ortholog of CXCL5, are expressed by human melanoma cells [44],[45].

Depletion of PMN-MDSC from 1 wk of age resulted in a decreased density of proliferating cells in primary tumors. Although no significant effect on tumor size was detected at 7 wk, when depletion was performed from 5 wk until 20 wk of age, primary tumors were much smaller. Importantly the size of cutaneous metastases, which contain few PMN-MDSC, was not affected by the depletion. Three sets of observations suggest that this effect is due to a direct action of PMN-MDSC on cancer cells. Firstly, highly purified PMN-MDSC stimulated the proliferation of Melan-Ret cells in vitro. This effect was mediated by soluble factors secreted by PMN-MDSC, since it did not require direct contact with the melanoma cells. Secondly, PMN-MDSC were the only tumor-infiltrating cells whose number was reduced by the antibody treatment; monocytes (CD11b^+^Gr1^lo^F4/80^lo^) were unaffected and macrophage (CD11b^+^Gr1^−^F4/80^+^) density was, if anything, slightly increased. Finally the number of endothelial cells (CD45^−^CD31^+^) was slightly increased in PMN-MDSC depleted tumors, showing that PMN-MDSC stimulated tumor growth independently of angiogenesis.

EMT has been suggested to be an essential step in cancer cell dissemination and metastasis [36]. Artificial induction of EMT in cancer cell lines promotes metastatic potential [46]. The aggressive behavior of melanoma has been attributed to its propensity to undergo EMT [47]. In the present study we show that purified PMN-MDSC induce typical features of EMT in vitro in melanoma and bladder human cell lines and in cells freshly isolated from primary and metastatic RETAAD tumors. In vivo, depletion of PMN-MDSC reduced the density of vimentin and S100A4 expressing cells in the primary tumor. Vimentin is a classical mesenchymal marker, while S100A4, a target of the EMT inducer Snail [48], is known to down-regulate E-Cadherin in mammary epithelial cell lines [49]. Collectively these observations indicate that PMN-MDSC play a crucial role in cancer cell EMT in vivo. Interestingly, expression of S100A4 correlates with shorter patient survival in several cancers, including melanoma [50].

PMN-MDSC-induced EMT results in multinodular development of the primary tumor, since mice depleted of PMN-MDSC had primary tumors composed of fewer nodules. This observation is in agreement with previous findings that multinodular growth requires cell motility [30],[31]. At 7 wk, decreased nodularity in PMN-MDSC-depleted tumors was observed in the absence of reduction in the whole tumor size, consistent with spatial redistribution of cancer cells, rather than inhibition of their development. In contrast, cutaneous tumors typically do not display such a multinodular structure, which is consistent with their low content of PMN-MDSC. In humans, many primary tumors exhibit higher fractal dimensions than normal tissue, as a result of their multinodular morphology. This morphology is thought to facilitate exponential growth of small avascular tumors, since in silico modeling indicates that the optimal tumor morphology under strong nutrient limitation is fractal [31],[51]. Therefore, induction of EMT in cancer cells is likely to be selected because it favors the growth of the primary tumor.

PMN-MDSC-induced EMT was also associated with cancer cell dissemination and metastatic outgrowth. Cancer cell dissemination was assessed in the regional lymph nodes and in the lungs by measuring *Dct* and *Mitf* expression. As shown previously, *Dct* expression correlates with the presence of S100B^+^ melanoma cells detected by IHC [5]. PMN-MDSC depletion from 1 wk of age resulted in reduced dissemination by as early as 7 wk. Reduced primary tumor burden could not explain the decreased cancer cell dissemination, since decreased expression of melanoma markers in the mandibular LN and the lungs preceded the effect on primary tumor size: no change in primary tumor size could be detected at 7 wk. Decreased *Dct* and *Mitf* expression after PMN-MDSC depletion could result from decreased influx of and/or decreased proliferation of cancer cells in the lungs. The latter might be expected because PMN-MDSC favor cancer cell proliferation and suppress CD8^+^ T cell activity. We cannot exclude any of these explanations. In fact RETAAD mice possess increased numbers of PMN-MDSC in their lungs when compared with non-transgenic littermates (unpublished data). Furthermore CD8^+^ T cells control the proliferation of disseminated cancer cells in the lungs [5]. However, in our experiments, *Dct* expression was measured at an early time point (7 wk of age) and lung micrometastases have a low mitotic index in untreated animals (1.9% + 0.5%; [5]). We calculated that even an 80% inhibition of cancer cell proliferation in the lungs could not account for the observed decrease in *Dct* expression (Text S1). Moreover, immunohistochemistry analysis of lungs from control and depleted mice at 7 wk revealed mostly individual cancer cells (CD45^-^S100B^+^) (Figure 5E). We therefore favor the interpretation that the decrease observed in *Dct* expression is mainly due to reduced colonization of the lungs by cancer cells rather than reduced proliferation.

Analysis of cutaneous metastases provided further support for decreased cancer cell dissemination in mice depleted of PMN-MDSC. Since PMN-MDSC poorly infiltrate cutaneous tumors, depletion was less likely to affect cancer cell proliferation in this site. If PMN-MDSC were acting by promoting metastatic growth rather than dissemination, we would expect to see smaller metastases in depleted mice. However, depletion of PMN-MDSC from 1 wk of age reduces the number of cutaneous tumors (Figure 5B), but not their size (Figure 5C). Interestingly, when PMN-MDSC depletion was started at 5 wk of age (i.e. 2–3 wk after the initiation of cancer cell dissemination), no significant change was seen in the number of cutaneous metastases (Figure 5D). This shows that metastatic potential had already been acquired at 5 wk and that cancer cells disseminating before 5 wk contain tumor initiating cells.

Depletion of PMN-MDSC resulted in a very significant inhibition of tumor cell dissemination (85% reduction in the draining lymph node, 74% in the lung, 40% reduction in the incidence of cutaneous tumors) and EMT (68% reduction in S100A4^+^ cells and 70% reduction in vimentin^+^ cells). Other tumor infiltrating immune cells have been shown to favor metastasis. In a mammary tumor model, CD11b^+^Gr1^+^ cells were shown to accumulate in the primary tumor and promote metastasis through increased production of MMP. Interestingly, in this model, EMT was not involved [52]. In a model of colorectal cancer, accumulation of immature myeloid cells distinct from MDSC favored invasion through the production of MMP [53]. PMN-MDSC from RETAAD tumors also produce MMP, mostly MMP9, but when RETAAD mice were crossed to an MMP9-KO background, no significant difference was observed in cancer cell dissemination or in the incidence of cutaneous metastases (unpublished data). Therefore, myeloid cells that infiltrate the primary tumor could contribute to metastasis through more than one mechanism.

Transcriptome analysis of the various cell subsets purified from RETAAD tumors showed that PMN-MDSC express TGF-β1 and HGF, whereas melanoma cells express EGF (Figure 7). This observation extends previous studies in a mammary tumor model showing that MDSC represent an abundant source of intratumoral TGF-β [52],[54]. We found that specific inhibitors of the corresponding receptors blocked PMN-MDSC mediated induction of EMT. The fact that EGF inhibitors reduced EMT induction while PMN-MDSC purified from RETAAD tumors did not produce EGF may suggest that PMN-MDSC stimulate EGF production by the cancer cells.

Cancer progression has been often depicted as a linear process, during which the incipient cancer cell sequentially accumulates genetic and epigenetic changes that confer the hallmarks of cancer cells [55]. Here we show that some of these properties can be induced by cues provided by the immune stroma of the primary tumor. Once the cell migrates out of the primary tumor, it may well lapse back to its original phenotype and, for example, undergo mesenchymal-epithelial transformation. Such a transient phenotypic switch may accelerate carcinogenesis and participate in the plasticity of cancer cells [56],[57].

Tumor infiltration by myeloid cells is associated with a poor prognosis for cancer patients. Most studies have focused on the role of macrophages [10],[58]–[61], whereas MDSC have been primarily characterized for their effects on immune cells [18],[62]–[66], stromal cells [67], or endothelial cells [68]. The present study demonstrates for the first time that PMN-MDSC act on cancer cells to promote proliferation, EMT, and dissemination. In turn, it provides a mechanistic explanation for the long-recognized link between inflammation and cancer progression.

## Materials and Methods

### Mice

Animal care and experimental procedures were approved by the IACUC (Application No. 090425) of the Biological Resource Center, 20 Biopolis Way, Singapore 138668. The generation of RETAAD mice has been previously described [69]. Rosa mT/mG reporter mice expressing tdTomato and *Il8rb-KO* mice were obtained for JAX Laboratories (Cat No. 007576 and 006848, respectively). *Il8rb-KO* mice were crossed with mice expressing EGFP under the Lysozyme promoter [70] to obtain Gr1^hi^ cells lacking CXCR2 and expressing EGFP.

### Antibodies

Anti-CD45-FITC, anti-CD19-PE, anti-CD11c-PerCP/Cy5.5, anti-Gr1-PE/Cy7, anti-F4/80-APC, anti-CD11b-APC/Cy7, anti-CD3-PE, anti-CD4-PerCP/Cy5.5, anti-CD8-PE/Cy7, anti-NK1.1-APC, and anti-CD45-PE antibodies were from Biolegend, and anti-I-A/I-E-eFluor450 and anti-CD45-eFluor450 antibodies were from eBioscience. Anti-Melanoma antibody (ab732) was obtained from Abcam. Anti-rat E-Cadherin antibody was from BD Pharmingen while anti-mouse E-cadherin antibody was from R&D Systems. Anti-mouse vimentin antibody was from Proteintech Group.

### Flow Cytometry

Tumors were dissected from RETAAD mice and single cell suspensions were obtained by digestion with Collagenase A (1 mg/ml; Roche) and DNase I (0.1 mg/ml, Roche) for 20 min at 37°C followed by filtration through a 70 µm filter (BD Biosciences). Flow cytometric data were acquired on the BD LSRII (BD Bioscience) and analyzed using FlowJo software (Tree Star, Inc.).

### Migration Assay

Dissociated tumor cells or recombinant CXCL1, CXCL2, and CXCL5 (R&D Systems) were placed in the bottom chamber of a 24-well plate, and 1×10^6^ total cells from the blood and spleen of 12-wk-old RETAAD mice were placed in the upper chamber (3 µm, BD Falcon). Cells were allowed to migrate to the bottom well for 3 h at 37°C, 5% CO_2_. Migrated cells were then analyzed by flow cytometry on the BD FACSCalibur and quantified using CountBright Absolute Counting Beads (Invitrogen). For the inhibition of CXCR2, PMN-MDSC were treated 1 h before and during the migration assay with inhibitors SB225002 and SB265610 from Tocris Bioscience.

For the in vivo migration assay, 5×10^6^ bone marrow cells from Rosa mT/mG reporter mice expressing tdTomato and an equal number of *Il8r-KO* bone marrow cells expressing GFP were injected intra-orbitally into tumor-bearing RETAAD mice. After 18 h, the ratio of tdTomato^+^ cells to GFP^+^ Gr1^hi^ cells infiltrating the contra-lateral tumor was measured by flow cytometry.

### qRT-PCR

For each mouse (*n* = 11), RNA was extracted from one primary tumor and one cutaneous tumor and gene expression was analyzed using Mouse Common Cytokines PCR Arrays (SABiosciences, PAMM-021) and Mouse Inflammatory Cytokines & Receptors PCR Arrays (SABiosciences, PAMM-011) following the manufacturer’s instructions. CXCL2 was analyzed separately using the following primers: 5’-AGTGAACTGCGCTGTCAATG-3’ and 5’-GAGAGTGGCTATGACTTCTGTCTG-3’. Gene expression was normalized to GAPDH expression, and *p* values were calculated using a two-tailed paired *t* test. *Dct* expression was measured as previously described [5].

### In Vivo PMN-MDSC Depletion

Mice were injected intraperitoneally twice a week with 0.25 mg of anti-Ly6G NIMP-R14 antibody [71] or control rat IgG (Sigma-Aldrich). Efficiency of PMN-MDSC depletion was monitored by flow cytometry. Mice were clinically assessed for palpable tumors once per fortnight. Injection schedules are as illustrated in Figure S3A.

### Immunohistochemistry

Formalin-fixed paraffin-embedded sections (5 µm) were immunolabeled for S100B and Ki67 as described previously [5] or for S100A4 (Abcam; ab27957). Tumor areas were assessed using ImagePro Analyzer 6.2 software (Media Cybernetics Inc.). Total cell number was calculated by dividing the tumor area by the average area of one cancer cell (estimated to be 82 µm^2^). The mitotic index was calculated as follows:
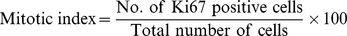



### Isolation of PMN-MDSC and Macrophages

PMN-MDSC and macrophages were isolated using an immune-magnetic separation kit—EasySep Mouse PE Positive Selection Kit (STEMCELL Technologies)—according to the manufacturer’s protocol. Briefly, PMN-MDSC and macrophages from the blood and spleen of 12-wk-old mice were labeled with anti-Ly6G-PE (1A8; BD Bioscience) or anti-F4/80-PE (BM8; eBioscience) antibodies, respectively. PE-labeled cells were immuno-magnetically labeled and positively selected using an EasySep magnet. Cell purity was verified by flow cytometry and was always >80%.

### OVA-Specific T Cell Proliferation Assay

CD8^+^ T cells were isolated from the spleen of C.Cg-Rag2^tm1Fwa^ Tg(DO11.10)10Dlo (Taconic #4219) using a CD8a^+^ T Cell Isolation Kit (Miltenyi Biotec). 1×10^5^ isolated T cells were plated with 2×10^4^ irradiated (2,000 rads) cells from the CD8^-^ fraction. PMN-MDSC isolated from tumor-bearing mice (12-wk-old) were added at a ratio of 1∶1, 1∶4, 1∶16, and 1∶64 to T cells. 5 µg/ml of SIINFEKL peptide (GL Biochem) was added. After 96 h incubation, T cell proliferation was measured by [^3^H]thymidine incorporation.

### EMT Assay

NBT-II cells, expressing histone H2B-mCherry, were seeded at a density of 100 cells per well on Ibidi 8 well µ-slides and left in culture for 4 d. Inhibitors were added on day 3. Purified PMN-MDSC (1×10^4^ cells/ml) were added on day 4 and left in co-culture for 24 h. EGF (100 ng/ml; Sigma) was used as a positive control. Inhibitors used were TGF-β1 receptor inhibitor, SB 525334 (Tocris; final concentration 10 µM), EGF receptor inhibitor, PD 153035 hydrochloride (Tocris; final concentration 8 µM), and HGF receptor inhibitor, JNJ38877605 (Selleck Chemicals; final concentration 5 µM). Time-lapse images were captured with an Olympus FV-1000 confocal system and analyzed with MacBiophotonics ImageJ [72] and Imaris (Bitplane). NBT-II cells were then fixed and stained with Phalloidin-Alexa Fluor 488 (Invitrogen) and mouse anti-rat Desmoplakin (Millipore; clone DP2.15). A goat anti-mouse antibody conjugated to Alexa Fluor 488 (Invitrogen) was used as a secondary antibody. Fluorescent images were captured with an Olympus IX-81 Inverted microscope and Retiva-SRV CCD Camera (QImaging) and analyzed with ImagePro analysis software (MediaCybernetics).

### Statistics

Gene expression data were log-transformed before analysis using Student’s *t* test. Statistical tests used to analyze other data are described in the individual figure legends. Prism (GraphPad Inc.) and Excel (Microsoft) softwares were used for calculations and graphing. *p* values less than 0.05 were considered statistically significant.

## Supporting Information

Figure S1 Accumulation of PMN-MDSC during melanoma progression and gating strategy. (A and B) PMN-MDSC were quantified by flow cytometry in the (A) spleen and (B) blood of RETAAD mice (RET^+^) and non-transgenic littermates (ret^−^). RETAAD mice displayed no sign of disease (Nil), exophthalmos (Ex), or macroscopic tumors (Tum). Graphs show the frequencies of CD11b^+^Gr1^hi^ cells among CD45^+^ cells. Bars represent mean ± SEM of four mice per group.*p value <0.05, **p value <0.01**.** (C) CD11b^+^Gr1^hi^ cells isolated from the spleen and blood of tumor-bearing mice (12-wk-old) inhibit T cell proliferation in a dose-dependent manner. Bars represent mean ± SEM; assay was performed in triplicate. (D) The figure shows the strategy used to enumerate myeloid cells in Figure 1: plasmacytoid dendritic cells (pDC), CD103^+^ and CD11b^+^ DC, PMN-MDSC, Mo-MDSC, and Macrophages. The bottom right panel shows May-Grünwald Giemsa staining of PMN-MDSC.
(TIF)

Figure S2 Expression of CXCR2 and its ligands in RETAAD tumors. CXCR2 (*Il8rb*), CXCL1, and CXCL2 are preferentially expressed in tumor-infiltrating PMN-MDSC, while CXCL5 is preferentially expressed by tumor cells. Data are from four individual experiments from sorted fractions of PMN-MDSCs and tumor cells. Bars represent mean ± SD.
(TIF)

Figure S3 Depletion of PMN-MDSC in RETAAD mice. (A) Schematic diagram illustrating the two depletion protocols used in this study. (B) Quantification of PMN-MDSC, Monocytic myeloid-derived suppressor cells (Mo-MDSC), and Macrophages in five individual tumors from mice injected with NIMP-R14 (Dp) and three individual tumors from mice injected with control (Ct) antibody (depletion scheme B). Bars represent mean ± SEM. *p value <0.05; ns, non-significant.
(TIF)

Figure S4 Depletion of PMN-MDSC does not affect the size of cutaneous metastases. Data are from 20-wk-old mice injected with control antibody (*n* = 6) or with anti-Ly6G antibody (*n* = 4) for 15 wk (depletion scheme B). Ns, non-significant, two-tailed *t* test.
(TIF)

Figure S5 PMN-MDSC depletion does not reduce vascularisation of the primary tumor. (A) Representative images of tumor vasculature (black arrows) in 7-wk-old-mice. No significant difference could be observed between the control and PMN-MDSC depleted samples. Depletion scheme A. (B) Quantification of endothelial cells (CD45^−^CD31^+^) by flow cytometry in five individual tumors from mice injected with NIMP-R14 (Dp) and three individual tumors from mice injected with control (Ct) antibody. Depletion scheme B. Bars represent mean ± SEM. Ns, non-significant.
(TIF)

Figure S6 Depletion of PMN-MDSC has no effect on the size of the primary tumor at 7 wk. Data are from 18 control and 22 PMN-MDSC depleted primary tumors (Depletion scheme A). Bars represent mean ± SEM. ns, non-significant.
(TIF)

Figure S7 Alternative Inhibitors block PMN-MDSC induced EMT. Alternative inhibitors block PMN-MDSC induced EMT in NBT-II cells. AG1478– EGFR inhibitor, SGX523– c-met (HGFR) inhibitor, and SB431542– TGF-βR1 inhibitor.
(TIF)

Table S1 Differential expression of chemokines and cytokines in primary tumors and metastases. Gene expression was measured by low density qRT-PCR arrays (except for *CXCL2* which was done by individual qRT-PCR). Expression values were normalized to *GAPDH* and presented as log_2_(expression in primary tumor/metastases). *p* values were calculated using two-tailed paired *t* test. Data are from 11 paired primary tumors and metastases. Each pair of primary and metastatic tumors is taken from the same individual mouse.
(DOC)

Text S1 Calculations used to estimate tumor size and *Dct* expression levels. Calculations were made to estimate the theoretical tumor size at 7 wk and 20 wk of age. Calculations were also made to estimate the theoretical decrease in *Dct* expression if cancer cell proliferation was inhibited by 80% in the lungs.
(DOC)

Video S1 Induction of EMT by PMN-MDSC. Videos of time lapse DIC images over 22 h displaying NBT-II cells without stimulation
(MOV)

Video S2 Induction of EMT by PMN-MDSC. Videos of time lapse DIC images over 22 h displaying NBT-II cells with EGF (100 ng/ml).
(MOV)

Video S3 Induction of EMT by PMN-MDSC. Videos of time lapse DIC images over 22 h displaying NBT-II cells with PMN-MDSC (1×10^4^ cells/well).
(MOV)

## Abbreviations

*Dct*: Daupachrome tautomerase
EGF: Epidermal Growth Factor
EMT: epithelial-mesenchymal transition
HGF: Hepatocyte Growth Factor
MDSC: myeloid-derived suppressor cell
TGF-β1: Transforming Growth Factor-β1

## References

[1] Jemal A, Siegel R, Ward E, Hao Y, Xu J (2008). Cancer statistics, 2008.. CA Cancer J Clin.

[2] Nguyen D. X, Bos P. D, Massague J (2009). Metastasis: from dissemination to organ-specific colonization.. Nat Rev Cancer.

[3] Fearon E. R, Vogelstein B (1990). A genetic model for colorectal tumorigenesis.. Cell.

[4] Bernards R, Weinberg R. A (2002). A progression puzzle.. Nature.

[5] Eyles J, Puaux A. L, Wang X, Toh B, Prakash C (2010). Tumor cells disseminate early, but immunosurveillance limits metastatic outgrowth, in a mouse model of melanoma.. J Clin Invest.

[6] Husemann Y, Geigl J. B, Schubert F, Musiani P, Meyer M (2008). Systemic spread is an early step in breast cancer.. Cancer Cell.

[7] Balkwill F, Mantovani A (2001). Inflammation and cancer: back to Virchow?. Lancet.

[8] Cordon-Cardo C, Prives C (1999). At the crossroads of inflammation and tumorigenesis.. J Exp Med.

[9] Grivennikov S. I, Greten F. R, Karin M (2010). Immunity, inflammation, and cancer.. Cell.

[10] Mantovani A (2010). Molecular pathways linking inflammation and cancer.. Curr Mol Med.

[11] DeNardo D. G, Andreu P, Coussens L. M (2010). Interactions between lymphocytes and myeloid cells regulate pro- versus anti-tumor immunity.. Cancer Metastasis Rev.

[12] Movahedi K, Laoui D, Gysemans C, Baeten M, Stange G (2010). Different tumor microenvironments contain functionally distinct subsets of macrophages derived from Ly6C(high) monocytes.. Cancer Res.

[13] Bronte V (2009). Myeloid-derived suppressor cells in inflammation: uncovering cell subsets with enhanced immunosuppressive functions.. Eur J Immunol.

[14] Gabrilovich D. I, Nagaraj S (2009). Myeloid-derived suppressor cells as regulators of the immune system.. Nat Rev Immunol.

[15] Ostrand-Rosenberg S, Sinha P (2009). Myeloid-derived suppressor cells: linking inflammation and cancer.. J Immunol.

[16] Ribechini E, Greifenberg V, Sandwick S, Lutz M. B (2010). Subsets, expansion and activation of myeloid-derived suppressor cells.. Med Microbiol Immunol.

[17] Peranzoni E, Zilio S, Marigo I, Dolcetti L, Zanovello P (2010). Myeloid-derived suppressor cell heterogeneity and subset definition.. Curr Opin Immunol.

[18] Movahedi K, Guilliams M, Van den Bossche J, Van den Bergh R, Gysemans C (2008). Identification of discrete tumor-induced myeloid-derived suppressor cell subpopulations with distinct T cell-suppressive activity.. Blood.

[19] Youn J. I, Nagaraj S, Collazo M, Gabrilovich D. I (2008). Subsets of myeloid-derived suppressor cells in tumor-bearing mice.. J Immunol.

[20] Yang L, Edwards C. M, Mundy G. R (2010). Gr-1+CD11b+ myeloid-derived suppressor cells: formidable partners in tumor metastasis.. J Bone Miner Res.

[21] Murdoch C, Muthana M, Coffelt S. B, Lewis C. E (2008). The role of myeloid cells in the promotion of tumour angiogenesis.. Nat Rev Cancer.

[22] Ostrand-Rosenberg S (2010). Myeloid-derived suppressor cells: more mechanisms for inhibiting antitumor immunity.. Cancer Immunol Immunother.

[23] Kato M, Takahashi M, Akhand A. A, Liu W, Dai Y (1998). Transgenic mouse model for skin malignant melanoma.. Oncogene.

[24] Eskelin S, Pyrhonen S, Summanen P, Hahka-Kemppinen M, Kivela T (2000). Tumor doubling times in metastatic malignant melanoma of the uvea: tumor progression before and after treatment.. Ophthalmology.

[25] Alla V, Engelmann D, Niemetz A, Pahnke J, Schmidt A (2010). E2F1 in melanoma progression and metastasis.. J Natl Cancer Inst.

[26] Thiery J. P, Acloque H, Huang R. Y, Nieto M. A (2009). Epithelial-mesenchymal transitions in development and disease.. Cell.

[27] Landsberg J, Gaffal E, Cron M, Kohlmeyer J, Renn M (2010). Autochthonous primary and metastatic melanomas in Hgf-Cdk4 R24C mice evade T-cell-mediated immune surveillance.. Pigment Cell Melanoma Res.

[28] Bronkhorst I. H, Ly L. V, Jordanova E. S, Vrolijk J, Versluis M (2011). Detection of M2-macrophages in uveal melanoma and relation with survival.. Invest Ophthalmol Vis Sci.

[29] Nagendra S, Schlueter A. J (2004). Absence of cross-reactivity between murine Ly-6C and Ly-6G.. Cytometry A.

[30] Narang V, Wong S. Y, Leong S. R, Abastado J. P, Gouaillard A Comparing mathematical models of cell adhesion in tumors; 2011; 2nd International Conference on Computational Systems - Biology and Bioinformatics..

[31] Ferreira S. C, Martins M. L, Vilela M. J (2002). Reaction-diffusion model for the growth of avascular tumor.. Phys Rev E Stat Nonlin Soft Matter Phys.

[32] Mallet D. G, De Pillis L. G (2006). A cellular automata model of tumor-immune system interactions.. J Theor Biol.

[33] Billottet C, Tuefferd M, Gentien D, Rapinat A, Thiery J. P (2008). Modulation of several waves of gene expression during FGF-1 induced epithelial-mesenchymal transition of carcinoma cells.. J Cell Biochem.

[34] Gavrilovic J, Moens G, Thiery J. P, Jouanneau J (1990). Expression of transfected transforming growth factor alpha induces a motile fibroblast-like phenotype with extracellular matrix-degrading potential in a rat bladder carcinoma cell line.. Cell Regul.

[35] Jouanneau J, Gavrilovic J, Caruelle D, Jaye M, Moens G (1991). Secreted or nonsecreted forms of acidic fibroblast growth factor produced by transfected epithelial cells influence cell morphology, motility, and invasive potential.. Proc Natl Acad Sci U S A.

[36] Thiery J. P (2002). Epithelial-mesenchymal transitions in tumour progression.. Nat Rev Cancer.

[37] Helfman D. M, Kim E. J, Lukanidin E, Grigorian M (2005). The metastasis associated protein S100A4: role in tumour progression and metastasis.. Br J Cancer.

[38] Grum-Schwensen B, Klingelhofer J, Berg C. H, El-Naaman C, Grigorian M (2005). Suppression of tumor development and metastasis formation in mice lacking the S100A4(mts1) gene.. Cancer Res.

[39] Boye K, Maelandsmo G. M (2010). S100A4 and metastasis: a small actor playing many roles.. Am J Pathol.

[40] Klein C. A (2009). Parallel progression of primary tumours and metastases.. Nat Rev Cancer.

[41] Melani C, Chiodoni C, Forni G, Colombo M. P (2003). Myeloid cell expansion elicited by the progression of spontaneous mammary carcinomas in c-erbB-2 transgenic BALB/c mice suppresses immune reactivity.. Blood.

[42] Eash K. J, Greenbaum A. M, Gopalan P. K, Link D. C (2010). CXCR2 and CXCR4 antagonistically regulate neutrophil trafficking from murine bone marrow.. J Clin Invest.

[43] Chintakuntlawar A. V, Chodosh J (2009). Chemokine CXCL1/KC and its receptor CXCR2 are responsible for neutrophil chemotaxis in adenoviral keratitis.. J Interferon Cytokine Res.

[44] Dhawan P, Richmond A (2002). Role of CXCL1 in tumorigenesis of melanoma.. J Leukoc Biol.

[45] Gutman M, Singh R. K, Xie K, Bucana C. D, Fidler I. J (1995). Regulation of interleukin-8 expression in human melanoma cells by the organ environment.. Cancer Res.

[46] Yang J, Weinberg R. A (2008). Epithelial-mesenchymal transition: at the crossroads of development and tumor metastasis.. Dev Cell.

[47] Gupta P. B, Kuperwasser C, Brunet J. P, Ramaswamy S, Kuo W. L (2005). The melanocyte differentiation program predisposes to metastasis after neoplastic transformation.. Nat Genet.

[48] Moody S. E, Perez D, Pan T. C, Sarkisian C. J, Portocarrero C. P (2005). The transcriptional repressor Snail promotes mammary tumor recurrence.. Cancer Cell.

[49] Keirsebilck A, Bonne S, Bruyneel E, Vermassen P, Lukanidin E (1998). E-cadherin and metastasin (mts-1/S100A4) expression levels are inversely regulated in two tumor cell families.. Cancer Res.

[50] Andersen K, Nesland J. M, Holm R, Florenes V. A, Fodstad O (2004). Expression of S100A4 combined with reduced E-cadherin expression predicts patient outcome in malignant melanoma.. Mod Pathol.

[51] Norton L (2005). Conceptual and practical implications of breast tissue geometry: toward a more effective, less toxic therapy.. Oncologist.

[52] Yang L, Huang J, Ren X, Gorska A. E, Chytil A (2008). Abrogation of TGF beta signaling in mammary carcinomas recruits Gr-1+CD11b+ myeloid cells that promote metastasis.. Cancer Cell.

[53] Kitamura T, Kometani K, Hashida H, Matsunaga A, Miyoshi H (2007). SMAD4-deficient intestinal tumors recruit CCR1+ myeloid cells that promote invasion.. Nat Genet.

[54] Bierie B, Moses H. L (2010). Transforming growth factor beta (TGF-beta) and inflammation in cancer.. Cytokine Growth Factor Rev.

[55] Cahill D. P, Kinzler K. W, Vogelstein B, Lengauer C (1999). Genetic instability and darwinian selection in tumours.. Trends Cell Biol.

[56] Chaffer C. L, Weinberg R. A (2011). A perspective on cancer cell metastasis.. Science.

[57] Ledford H (2011). Cancer theory faces doubts.. Nature.

[58] Qian B. Z, Pollard J. W (2010). Macrophage diversity enhances tumor progression and metastasis.. Cell.

[59] Lin E. Y, Nguyen A. V, Russell R. G, Pollard J. W (2001). Colony-stimulating factor 1 promotes progression of mammary tumors to malignancy.. J Exp Med.

[60] Robinson B. D, Sica G. L, Liu Y. F, Rohan T. E, Gertler F. B (2009). Tumor microenvironment of metastasis in human breast carcinoma: a potential prognostic marker linked to hematogenous dissemination.. Clin Cancer Res.

[61] Doedens A. L, Stockmann C, Rubinstein M. P, Liao D, Zhang N (2010). Macrophage expression of hypoxia-inducible factor-1{alpha} suppresses T-cell function and promotes tumor progression.. Cancer Res.

[62] Greifenberg V, Ribechini E, Rossner S, Lutz M. B (2009). Myeloid-derived suppressor cell activation by combined LPS and IFN-gamma treatment impairs DC development.. Eur J Immunol.

[63] Sinha P, Clements V. K, Bunt S. K, Albelda S. M, Ostrand-Rosenberg S (2007). Cross-talk between myeloid-derived suppressor cells and macrophages subverts tumor immunity toward a type 2 response.. J Immunol.

[64] Marigo I, Bosio E, Solito S, Mesa C, Fernandez A (2010). Tumor-induced tolerance and immune suppression depend on the C/EBPbeta transcription factor.. Immunity.

[65] Hanson E. M, Clements V. K, Sinha P, Ilkovitch D, Ostrand-Rosenberg S (2009). Myeloid-derived suppressor cells down-regulate L-selectin expression on CD4+ and CD8+ T cells.. J Immunol.

[66] Corzo C. A, Cotter M. J, Cheng P, Cheng F, Kusmartsev S (2009). Mechanism regulating reactive oxygen species in tumor-induced myeloid-derived suppressor cells.. J Immunol.

[67] Rosenberg S. A, Yang J. C, Restifo N. P (2004). Cancer immunotherapy: moving beyond current vaccines.. Nat Med.

[68] Yang L, DeBusk L. M, Fukuda K, Fingleton B, Green-Jarvis B (2004). Expansion of myeloid immune suppressor Gr+CD11b+ cells in tumor-bearing host directly promotes tumor angiogenesis.. Cancer Cell.

[69] Lengagne R, Graff-Dubois S, Garcette M, Renia L, Kato M (2008). Distinct role for CD8 T cells toward cutaneous tumors and visceral metastases.. J Immunol.

[70] Faust N, Varas F, Kelly L. M, Heck S, Graf T (2000). Insertion of enhanced green fluorescent protein into the lysozyme gene creates mice with green fluorescent granulocytes and macrophages.. Blood.

[71] Lopez A. F, Strath M, Sanderson C. J (1984). Differentiation antigens on mouse eosinophils and neutrophils identified by monoclonal antibodies.. Br J Haematol.

[72] Abramoff M. D, Magelhaes P. J, Ram S. J (2004). Image processing with ImageJ.. Biophotonics International.

