# Supplementary figures and images for: Mesenchymal Transition and Dissemination of Cancer Cells Is Driven by Myeloid-Derived Suppressor Cells Infiltrating the Primary Tumor
Source: PLoS Biol. 2011 Sep 27;9(9):e1001162. doi: 10.1371/journal.pbio.1001162 (PMC3181226; doi:10.1371/journal.pbio.1001162)

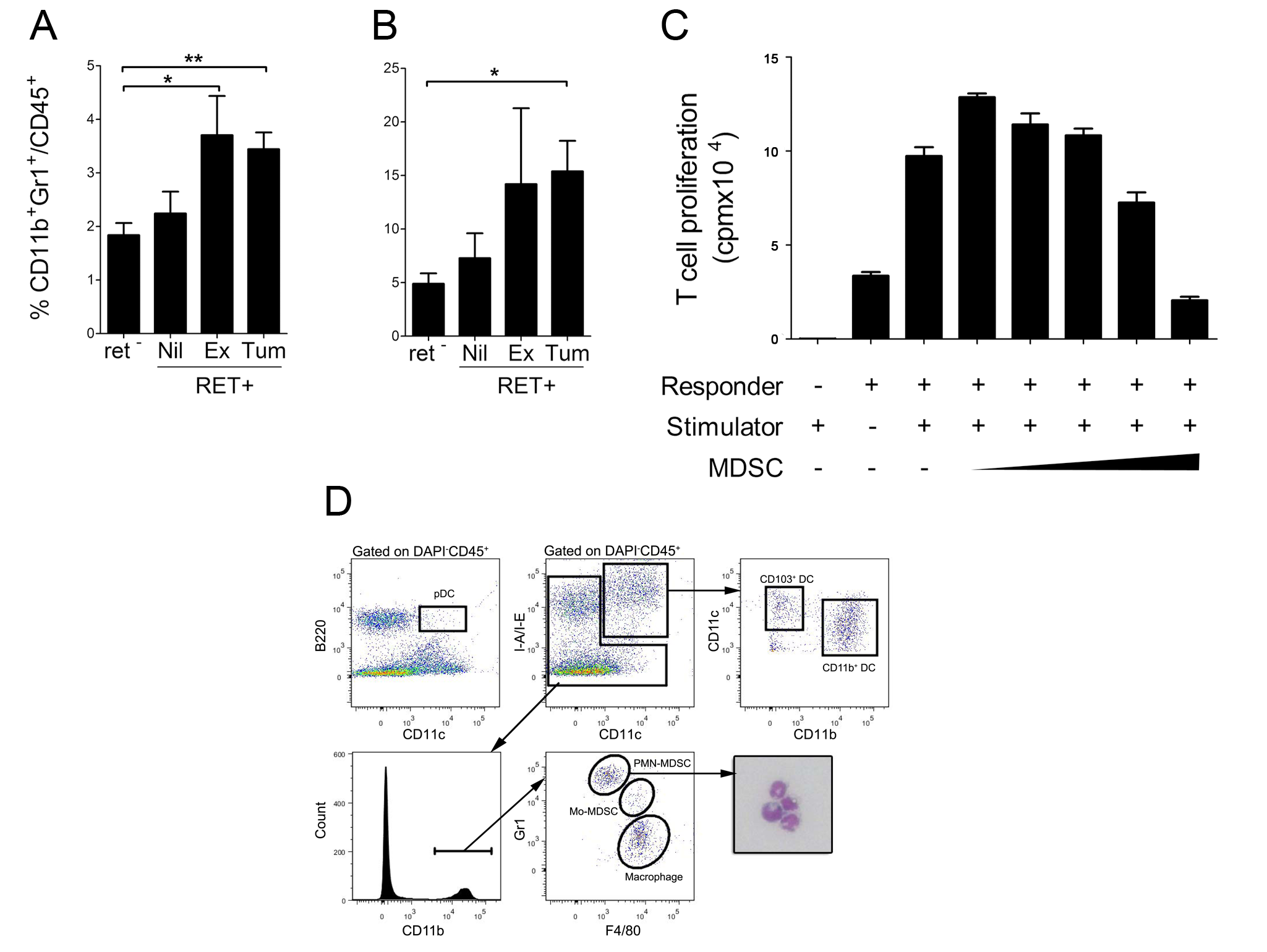

Supplement: Figure S1 — Accumulation of PMN-MDSC during melanoma progression and gating strategy. (A and B) PMN-MDSC were quantified by flow cytometry in the (A) spleen and (B) blood of RETAAD mice (RET+) and non-transgenic littermates (ret−). RETAAD mice displayed no sign of disease (Nil), exophthalmos (Ex), or macroscopic tumors (Tum). Graphs show the frequencies of CD11b+Gr1hi cells among CD45+ cells. Bars represent mean ± SEM of four mice per group.*p value <0.05, **p value <0.01. (C) CD11b+Gr1hi cells isolated from the spleen and blood of tumor-bearing mice (12-wk-old) inhibit T cell proliferation in a dose-dependent manner. Bars represent mean ± SEM; assay was performed in triplicate. (D) The figure shows the strategy used to enumerate myeloid cells in Figure 1: plasmacytoid dendritic cells (pDC), CD103+ and CD11b+ DC, PMN-MDSC, Mo-MDSC, and Macrophages. The bottom right panel shows May-Grünwald Giemsa staining of PMN-MDSC. (TIF) [file pbio.1001162.s001.tif]

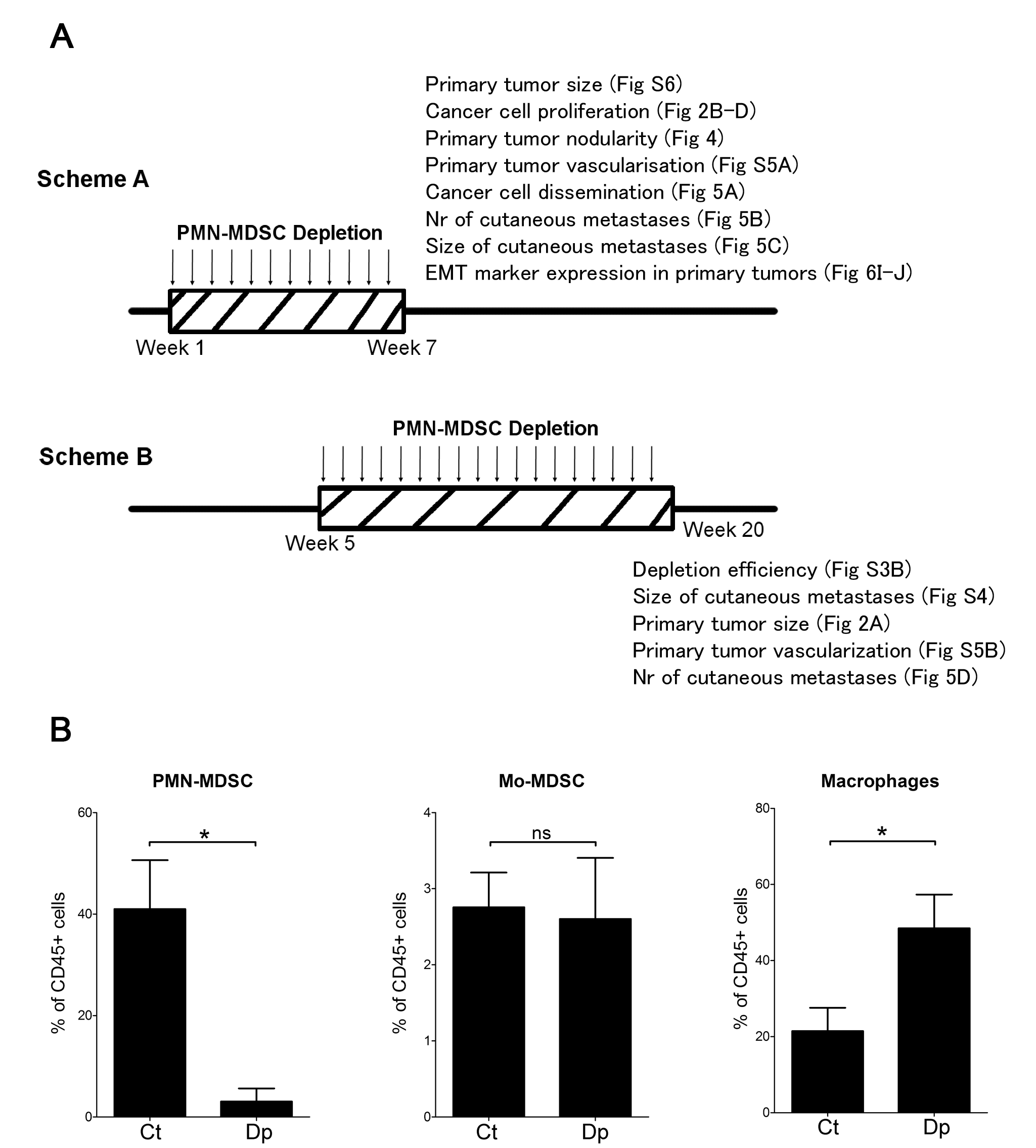

Supplement: Figure S3 — Depletion of PMN-MDSC in RETAAD mice. (A) Schematic diagram illustrating the two depletion protocols used in this study. (B) Quantification of PMN-MDSC, Monocytic myeloid-derived suppressor cells (Mo-MDSC), and Macrophages in five individual tumors from mice injected with NIMP-R14 (Dp) and three individual tumors from mice injected with control (Ct) antibody (depletion scheme B). Bars represent mean ± SEM. *p value <0.05; ns, non-significant. (TIF) [file pbio.1001162.s003.tif]

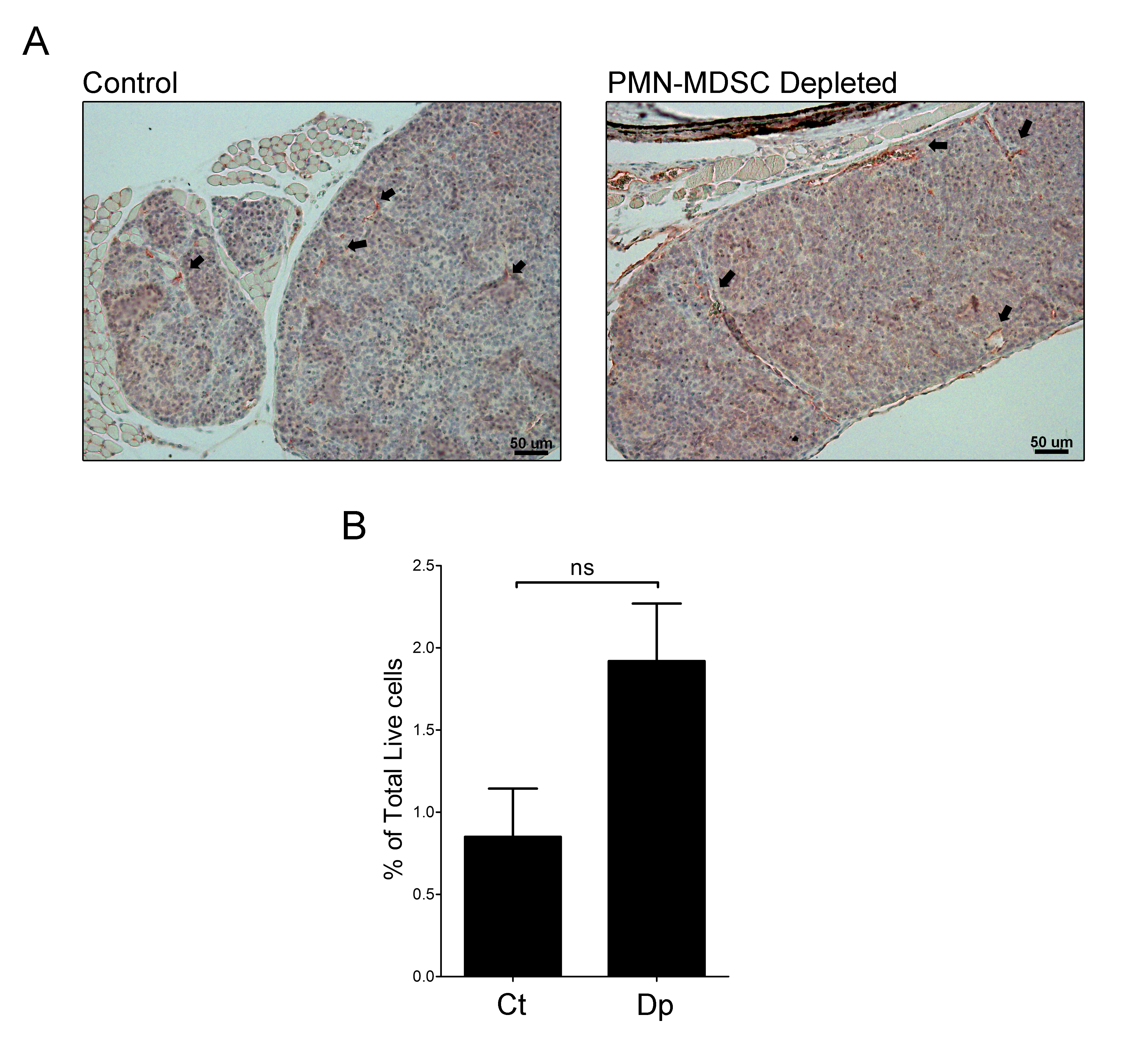

Supplement: Figure S5 — PMN-MDSC depletion does not reduce vascularisation of the primary tumor. (A) Representative images of tumor vasculature (black arrows) in 7-wk-old-mice. No significant difference could be observed between the control and PMN-MDSC depleted samples. Depletion scheme A. (B) Quantification of endothelial cells (CD45−CD31+) by flow cytometry in five individual tumors from mice injected with NIMP-R14 (Dp) and three individual tumors from mice injected with control (Ct) antibody. Depletion scheme B. Bars represent mean ± SEM. Ns, non-significant. (TIF) [file pbio.1001162.s005.tif]

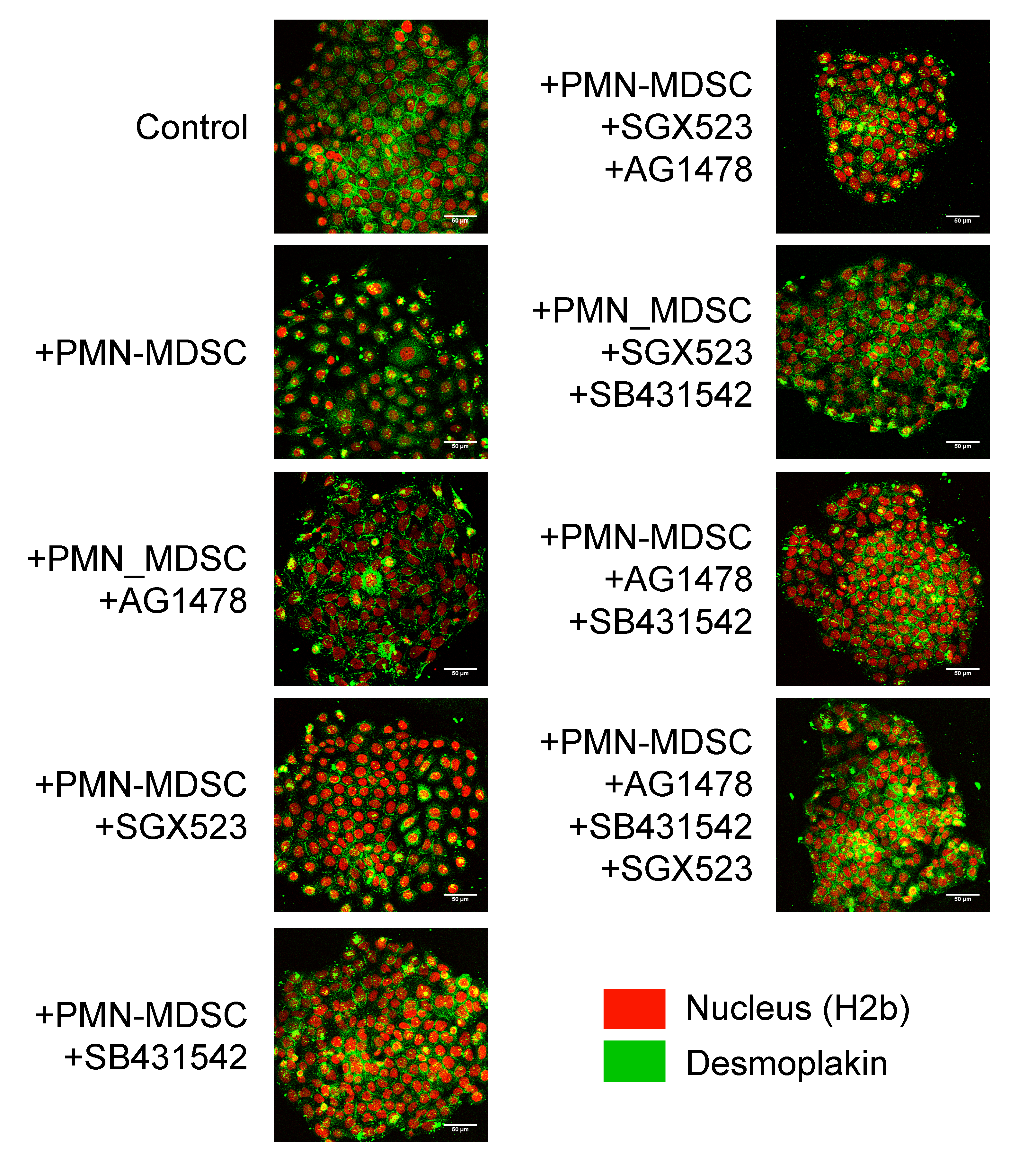

Supplement: Figure S7 — Alternative Inhibitors block PMN-MDSC induced EMT. Alternative inhibitors block PMN-MDSC induced EMT in NBT-II cells. AG1478– EGFR inhibitor, SGX523– c-met (HGFR) inhibitor, and SB431542– TGF-βR1 inhibitor. (TIF) [file pbio.1001162.s007.tif]
