# Supplementary material for: Mesenchymal Transition and Dissemination of Cancer Cells Is Driven by Myeloid-Derived Suppressor Cells Infiltrating the Primary Tumor
Source: PLoS Biol. 2011 Sep 27;9(9):e1001162. doi: 10.1371/journal.pbio.1001162 (PMC3181226; doi:10.1371/journal.pbio.1001162)
Supplement: Table S1 — Differential expression of chemokines and cytokines in primary tumors and metastases. Gene expression was measured by low density qRT-PCR arrays (except for CXCL2 which was done by individual qRT-PCR). Expression values were normalized to GAPDH and presented as log2(expression in primary tumor/metastases). p values were calculated using two-tailed paired t test. Data are from 11 paired primary tumors and metastases. Each pair of primary and metastatic tumors is taken from the same individual mouse. (DOC) [file pbio.1001162.s008.doc]

**Table S1: Differential expression of chemokines and cytokines in primary tumors and metastases**

| **Gene** | **Log_2_ Fold Change** | **p-value** |  | **Gene** | **Log_2_ Fold Change** | **p-value** |  | **Gene** | **Log_2_ Fold Change** | **p-value** |
| --- | --- | --- | --- | --- | --- | --- | --- | --- | --- | --- |
| **Abcf1** | 0.66 | 0.036 |  | **Cxcl11** | 0.533 | 0.415 |  | **Il20** | 1.919 | 0.041 |
| **Bcl6** | 0.497 | 0.122 |  | **Cxcl12** | 0.477 | 0.176 |  | **Il21** | 1.151 | 0.350 |
| **Bmp1** | 0.913 | 0.035 |  | **Cxcl13** | 0.646 | 0.048 |  | **Il24** | 1.146 | 0.340 |
| **Bmp10** | 0.674 | 0.539 |  | **Cxcl15** | 0.879 | 0.015 |  | **Il25** | -0.216 | 0.796 |
| **Bmp2** | -0.501 | 0.393 |  | **Cxcl2** | 2.163 | 0.037 |  | **Il27** | 0.224 | 0.846 |
| **Bmp3** | 0.653 | 0.322 |  | **Cxcl5** | 5.805 | 8.385E-05 |  | **Il2rb** | 0.012 | 0.969 |
| **Bmp4** | 1.815 | 0.153 |  | **Cxcl9** | -0.586 | 0.205 |  | **Il2rg** | 0.357 | 0.056 |
| **Bmp5** | -1.373 | 0.049 |  | **Cxcr3** | 0.53 | 0.074 |  | **Il3** | 0.361 | 0.330 |
| **Bmp6** | 0.592 | 0.118 |  | **Cxcr5** | 0.671 | 0.145 |  | **Il4** | 0.692 | 0.160 |
| **Bmp7** | -0.174 | 0.836 |  | **Fasl** | 0.014 | 0.977 |  | **Il5ra** | 0.667 | 0.065 |
| **Bmp8b** | 0.823 | 0.177 |  | **Fbrs** | -0.053 | 0.866 |  | **Il6ra** | 0.349 | 0.508 |
| **C3** | -0.276 | 0.705 |  | **Fgf10** | 0.04 | 0.963 |  | **Il6st** | 0.452 | 0.021 |
| **Casp1** | 0.493 | 0.410 |  | **Flt3l** | 0.072 | 0.803 |  | **Il7** | 0.891 | 0.025 |
| **Ccl1** | 0.481 | 0.153 |  | **Gdf1** | 0.528 | 0.183 |  | **Il8rb** | 1.659 | 0.013 |
| **Ccl11** | 0.46 | 0.460 |  | **Gdf10** | 1.476 | 0.080 |  | **Il9** | 0.386 | 0.708 |
| **Ccl12** | 1.162 | 0.014 |  | **Gdf11** | 0.168 | 0.675 |  | **Inha** | 0.985 | 0.060 |
| **Ccl17** | 0.801 | 0.151 |  | **Gdf15** | -0.328 | 0.510 |  | **Inhba** | 0.846 | 0.061 |
| **Ccl19** | 2.405 | 0.003 |  | **Gdf2** | 0.495 | 0.378 |  | **Itgam** | 0.884 | 0.001 |
| **Ccl2** | 0.325 | 0.318 |  | **Gdf3** | 0.738 | 0.275 |  | **Itgb2** | 0.626 | 0.017 |
| **Ccl20** | 0.989 | 0.004 |  | **Gdf5** | 0.536 | 0.512 |  | **Lefty1** | 0.37 | 0.613 |
| **Ccl22** | 0.367 | 0.577 |  | **Gdf9** | 0.428 | 0.446 |  | **Lif** | 0.989 | 0.117 |
| **Ccl24** | -0.004 | 0.994 |  | **Ifna2** | 0.464 | 0.480 |  | **Lta** | 0.279 | 0.542 |
| **Ccl25** | 0.761 | 0.093 |  | **Ifna4** | -0.216 | 0.712 |  | **Ltb** | 0.882 | 0.116 |
| **Ccl3** | 0.92 | 0.115 |  | **Ifnb1** | -0.762 | 0.284 |  | **Mif** | 0.221 | 0.065 |
| **Ccl4** | 0.366 | 0.611 |  | **Ifng** | 0.399 | 0.412 |  | **Mstn** | -1.548 | 0.182 |
| **Ccl5** | 0.249 | 0.185 |  | **Il10** | -0.286 | 0.579 |  | **Pf4** | -0.426 | 0.362 |
| **Ccl6** | 0.277 | 0.530 |  | **Il10ra** | 0.489 | 0.012 |  | **Scgb3a1** | -0.306 | 0.532 |
| **Ccl7** | 0.486 | 0.277 |  | **Il10rb** | 0.675 | 0.043 |  | **Scye1** | 0.689 | 0.011 |
| **Ccl8** | -0.108 | 0.859 |  | **Il11** | 0.766 | 0.032 |  | **Spp1** | 1.238 | 0.207 |
| **Ccl9** | 0.477 | 0.205 |  | **Il12b** | 0.491 | 0.646 |  | **Tgfb1** | 0.167 | 0.358 |
| **Ccr1** | 0.966 | 0.042 |  | **Il13** | 0.864 | 0.039 |  | **Tnf** | 1.336 | 0.026 |
| **Ccr10** | 0.672 | 0.057 |  | **Il13ra1** | 1.323 | 0.001 |  | **Tnfrsf11b** | 0.188 | 0.854 |
| **Ccr2** | 0.032 | 0.932 |  | **Il15** | 0.762 | 0.045 |  | **Tnfrsf1a** | 0.412 | 0.003 |
| **Ccr3** | 0.53 | 0.114 |  | **Il16** | 1.001 | 0.003 |  | **Tnfrsf1b** | 1.003 | 0.027 |
| **Ccr4** | 0.225 | 0.510 |  | **Il17b** | 0.388 | 0.311 |  | **Tnfsf10** | 1.62 | 4.298E-04 |
| **Ccr5** | 0.853 | 0.023 |  | **Il17c** | 1.861 | 0.051 |  | **Tnfsf11** | 0.814 | 0.554 |
| **Ccr6** | 0.642 | 0.033 |  | **Il17f** | 0.323 | 0.295 |  | **Tnfsf12** | -0.605 | 0.595 |
| **Ccr7** | 0.738 | 0.091 |  | **Il18** | 0.939 | 0.046 |  | **Tnfsf13** | -0.174 | 0.740 |
| **Ccr8** | 0.649 | 0.163 |  | **Il19** | -0.134 | 0.842 |  | **Tnfsf13b** | 1.504 | 0.354 |
| **Ccr9** | 0.42 | 0.339 |  | **Il1a** | 1.793 | 0.002 |  | **Tnfsf14** | 1.602 | 0.234 |
| **Cd40lg** | 0.612 | 0.069 |  | **Il1b** | 2.851 | 1.872E-04 |  | **Tnfsf15** | 2.042 | 0.331 |
| **Cd70** | 0.086 | 0.935 |  | **Il1f10** | -0.049 | 0.924 |  | **Tnfsf18** | 0.408 | 0.697 |
| **Crp** | 0.542 | 0.136 |  | **Il1f5** | 1.164 | 0.277 |  | **Tnfsf4** | -0.359 | 0.581 |
| **Csf1** | 0.524 | 0.213 |  | **Il1f6** | 3.463 | 0.001 |  | **Tnfsf8** | 0.394 | 0.363 |
| **Csf2** | 0.97 | 0.119 |  | **Il1f8** | 3.158 | 0.011 |  | **Tnfsf9** | 1.19 | 0.072 |
| **Ctf1** | 0.508 | 0.206 |  | **Il1f9** | 5.067 | 0.001 |  | **Tollip** | 0.374 | 0.121 |
| **Ctf2** | 0.386 | 0.458 |  | **Il1r1** | 1.258 | 0.003 |  | **Txlna** | -0.339 | 0.779 |
| **Cx3cl1** | 0.886 | 0.002 |  | **Il1r2** | 2.742 | 0.014 |  | **Xcr1** | -0.096 | 0.860 |
| **Cxcl1** | 2.663 | 3.403E-05 |  | **Il1rn** | 3.606 | 0.001 |  |  |  |  |
| **Cxcl10** | -0.474 | 0.363 |  | **Il2** | 0.025 | 0.982 |  |  |  |  |
