# Supplementary material for: Mesenchymal Transition and Dissemination of Cancer Cells Is Driven by Myeloid-Derived Suppressor Cells Infiltrating the Primary Tumor
Source: PLoS Biol. 2011 Sep 27;9(9):e1001162. doi: 10.1371/journal.pbio.1001162 (PMC3181226; doi:10.1371/journal.pbio.1001162)
Supplement: Text S1 — Calculations used to estimate tumor size and Dct expression levels. Calculations were made to estimate the theoretical tumor size at 7 wk and 20 wk of age. Calculations were also made to estimate the theoretical decrease in Dct expression if cancer cell proliferation was inhibited by 80% in the lungs. (DOC) [file pbio.1001162.s009.doc]

**Effect of reduced mitotic index on tumor diameter:**

At early time points, tumor growth is assumed to be exponential and described by the following equation (1):

(1) N= N_0_exp (kt/τ),

Where N is the number of melanoma cells at the end of the experiment, N_0_ is the number of melanoma cells at the onset of the experiment, k is the fraction of proliferating cells, t is the duration of the experiment (6 or 19 weeks), τ is the duration of the cell cycle (28 hrs, according to Gordon 1980, *Canc Res 40, 4467-4472).*

The ratio N_c_/N_d_ between the tumor mass in control vs depleted mice is given by:

(2) N_c_/N_d_= exp(k_c_-k_d_)t/τ)

Where k_c_ is the fraction of proliferating cells in the tumor of control mice and k_d_ is the fraction of proliferating cells in the tumor of PMN-MDSC-depleted mice.

For tumors with ellipsoid shape, the longer diameter is proportional to the cubic root of the cell number. If D_c_ is the average tumor diameter in control mice and D_d_, the average tumor diameter in depleted mice, their ratio is given by:

(3)D_c_/D_d_= [exp(k_c_-k_d_)t/τ)]^1/3^

k_c_ and k_d_ are deduced from MI, the mitotic index defined as the fraction of Ki67^+^ cells, and S, the duration of Ki67 expression during cell cycle (estimated to be 14h based on Lopez 1991 *Cytometry 12:42-49*) according to:

(4) k = MI.τ/S

From (3) and (4), one gets:

(5) D_c_/D_d_= [exp(MI_c_-MI_d_)t/S)]^1/3^

We measured MI_c_=0.035 and MI_d_=0.017.

At 7 weeks of age, the expected D_c_/D_d_ was 1.7 and no significant difference could be detected experimentally.

At 20 weeks of age, the expected D_c_/D_d_ was 5, in good agreement with the 4.7 value measured experimentally.

It should be noted that while some parameters have been experimentally measured, others were drawn from the literature. Therefore, one should keep in mind that these calculations are speculative.

**Decreased ectopic expression of *Dct* is not due to reduced cancer cell proliferation**

At early time points, the population of melanoma cells in the lung can be described by the following equation (1):

(1) N= N_0_exp (kt/τ),

Where N is the number of melanoma cells at the end of the experiment, N_0_ is the number of melanoma cells at the onset of the experiment, k is the fraction of proliferating cells, t is the duration of the experiment (6 weeks), τ is the duration of the cell cycle (28 hrs, according to Gordon 1980, *Canc Res 40, 4467-4472).*

It is assumed that *Dct* expression is proportional to N. Therefore,

(2) Dct_c_/Dct_d_= exp((k_c_-k_d_)t/τ)

Where Dct_c_/Dct_d_ is the ratio of *Dct* expression in the lungs of control vs PMN-MDSC-depleted mice, k_c_ is the fraction of proliferating cells in the lungs of control mice and k_d_ is the fraction of proliferating cells in the lungs of PMN-MDSC-depleted mice.

(3) k_c_ = MI_c_ τ/S

where MI_c_ is the mitotic index (the fraction of Ki67^+^ cells) in the lung of control animals (1.9%, see Eyles et al. 2010 *J. Clin. Invest.),* S is the duration of Ki67 expression during the cell cycle (estimated to be 14h based on Lopez 1991 *Cytometry 12:42-49*)

We tested the hypothesis that PMN-MDSC depletion would reduce the proliferation of disseminated cancer cells by 80%. Therefore

(4) k_d_= 0.2 k_c_

By combining (2), (3) and (4), one finds

(5) Dct_c_/Dct_d_=3

Therefore, even an 80% inhibition of cancer cell proliferation in the lungs could not account for the observed 5.5 fold decrease in *Dct* expression. We therefore favor the interpretation that the decrease observed in *Dct* expression is mainly due to reduced colonization of the lungs by cancer cells rather than reduced proliferation. It should be noted that while some parameters have been experimentally measured, others were drawn from the literature. Therefore, one should keep in mind that these calculations are speculative.
